# Effectiveness and acceptability of cognitive–behavioural therapy delivery formats for obsessive–compulsive disorder: network meta-analysis

**DOI:** 10.1192/bjp.2024.197

**Published:** 2026-03

**Authors:** Yingying Wang, Clara Miguel, Marketa Ciharova, Arpana Amarnath, Jingyuan Lin, Ruiying Zhao, Marieke B. J. Toffolo, Mathias Harrer, Sascha Y. Struijs, Leonore M. de Wit, Pim Cuijpers

**Affiliations:** Department of Clinical, Neuro and Developmental Psychology, Amsterdam Public Health Research Institute, Vrije Universiteit Amsterdam, Amsterdam, The Netherlands; The Institute of Brain and Psychological Science, Sichuan Normal University, Chengdu, China; Psychology & Digital Mental Health Care, Technical University Munich, Munich, Germany; International Institute for Psychotherapy, Babeș-Bolyai University, Cluj-Napoca, Romania

**Keywords:** Obsessive–compulsive disorders, network meta-analyses, cognitive–behavioural therapy, cognitive–behavioural therapy delivery format, effectiveness and acceptability

## Abstract

**Background:**

While various delivery formats of cognitive–behavioural therapy (CBT) for obsessive–compulsive disorder (OCD) are available, comprehensive evidence on their comparative effectiveness and acceptability is lacking.

**Aim:**

To examine the comparative effectiveness and acceptability of different CBT delivery formats for OCD.

**Method:**

An existing database of psychological interventions for OCD was utilised, with randomised controlled trials (RCTs) comparing CBT delivery formats with each other/control groups were included. Pairwise and network meta-analyses were conducted using a random-effects model. Comparative standard mean differences (SMDs) were calculated for effectiveness in reducing OCD symptom severity post-treatment. Relative risks were calculated for acceptability (conceptualised as any cause discontinuation in the acute treatment phase).

**Results:**

A total of 61 RCTs involving 3710 patients with OCD were included. All CBT treatment formats were significantly more effective than control groups (SMDs: −0.39 to −1.66). No significant differences were found among individual, remote-delivery, guided self-help, time-intensive and family-involved formats. However, individual, remote-delivery and family-involved formats were more effective than group (SMDs, −0.38 to −0.60), and most treatment formats were more effective than unguided self-help (SMDs, −0.58 to −0.80). Regarding acceptability, most CBT formats showed no significant differences among themselves, although they were generally more acceptable (relative risks: 1.11–1.18) than unguided self-help.

**Conclusions:**

Most CBT delivery formats serve as potential alternatives to conventional individual CBT. Unguided self-help has lower but still moderate effects in reducing OCD symptom severity, and it holds important potential for assisting a larger number of individuals with OCD who face barriers to accessing treatments.

Obsessive–compulsive disorder (OCD) is a chronic and debilitating mental disorder,^1^ adversely affecting both individual patients and their families.^2,3^ Cognitive–behavioural therapy (CBT) stands as the gold standard for treating OCD, traditionally delivered during individual, face-to-face weekly sessions. However, alternative CBT delivery formats have also shown effectiveness in OCD treatment. These formats include remote-delivery,^4–7^ group,^8–10^ guided self-help,^11–13^ unguided self-help,^14–16^ family-involved^17–19^ and time-intensive formats.^20–22^ Prior conventional pairwise meta-analyses have examined the effectiveness of these CBT delivery formats,^23–26^ including direct comparisons (i.e. examined in the same trial) between specific delivery formats, such as individual versus group formats.^27–29^ However, these analyses are limited by their focus on direct evidence from head-to-head comparisons within individual studies. They often exclude indirect evidence, which is obtained by comparing treatments via a common comparator. This indirect evidence is crucial for drawing inferences about the relative effectiveness of treatments that have not been directly compared in the same study, thereby enhancing the overall understanding of the field.

## Study aims and hypothesis

Network meta-analyses (NMAs) overcome the limitations of conventional meta-analyses by making optimal use of all available evidence by combining both direct and indirect evidence. They not only provide a comprehensive estimate of effectiveness, but also enable the ranking of treatments to identify the most and least effective among them.^30^ A recent NMA^31^ on paediatric OCD explored the efficacy and acceptability of in-person CBT, telephone/webcam CBT and internet-delivered CBT, finding that in-person CBT was significantly more effective than internet-delivered CBT, but not significantly different from telephone/webcam CBT. However, to the best of our knowledge, no study has simultaneously investigated the effectiveness and acceptability of all available CBT delivery formats for OCD in both paediatric and adult populations. Addressing this question is crucial for individuals with OCD and the future organisation and optimisation of mental healthcare system resources. Therefore, our objective was to conduct a new NMA to investigate the effectiveness and acceptability of various CBT delivery formats for OCD. Drawing on existing knowledge,^4,25,26,32–35^ we hypothesised that all CBT delivery formats would be significantly more effective than control groups, with no specific expectations regarding acceptability.

## Method

### Identification and selection of studies

We utilised a database of psychological interventions for OCD, which was built through comprehensive searches in both international (PubMed, Embase, PsycINFO, international clinical trials registry platform (ICTRP) of the World Health Organization (WHO)) and Chinese (China National Knowledge Infrastructure (CNKI), WeiPu, WanFang, Chinese Clinical Trial Registry (CCTR)) databases, spanning from inception to 1 September 2023. China, as the second most populous country globally, has a substantial number of individuals suffering from OCD.^36^ However, while many Chinese researchers have explored OCD,^37–39^ their work might not be accessible in international databases owing to language barriers. We have already published two meta-analyses with this database.^32,33^ Detailed search strings can be found in Supplementary Appendix A available at https://doi.org/10.1192/bjp.2024.197. Data extraction commenced on 11 September 2023. The study protocol was prospectively registered at the Open Science Framework (https://archive.org/details/osf-registrations-54cm7-v1).

Randomised controlled trials (RCTs) that compared a CBT delivery format to a control group or another CBT delivery format for individuals with a primary diagnosis of OCD, using valid clinical semi-structured interviews, were included. There were no restrictions on the duration of treatment, the number of sessions or the minimum number of participants. In addition, no limitations were placed on gender, age or setting.

Trials designed as stepped-care management, maintenance treatment or relapse prevention were excluded, as were comparisons of one certain CBT delivery format with only slight differences in treatment content.

We defined a treatment as CBT when it contained an active component of CBT, including exposure and response prevention (ERP) only, cognitive therapy only, ERP + cognitive therapy and third-wave CBT (e.g. acceptance and commitment therapy). The CBT delivery formats encompassed individual (face-to-face weekly sessions only), remote delivery (similar to individual CBT but conducted via technology aids such as telephone or videoconferencing), group, guided self-help, unguided self-help, family involved (involving the patient's family members in treatment sessions) and time intensive (a maximum total duration of 4 weeks, requiring a minimum of ten therapist hours overall^26^). More extensive definitions of formats can be found in Supplementary Appendix B. Control groups included waitlist, care-as-usual, psychological placebo and pill placebo.

All records were screened and selected by two independent researchers, with any disagreements, such as the rating of the delivery format, resolved through discussion or by a third, senior researcher.

### Data extraction and quality assessment

We extracted data across three domains: (a) characteristics of participants, such as mean age, proportion of female and proportion of participants using psychiatric medication; (b) characteristics of the intervention, such as the type of treatment format, whether the treatment was administered by trained therapists and treatment integrity implementation; (c) characteristics of the study, such as region of study origin and recruitment method of participants.

The revised Cochrane risk-of-bias tool for randomised trials (RoB 2)^40^ was used to assess the validity of the included studies. Biases were considered across five domains: (a) randomisation process, (b) deviations from intended interventions, (c) missing outcome data, (d) outcome measurement (e) and selective outcome reporting.

Data extraction and quality assessment were conducted by two independent researchers, and any disagreements were solved through discussion.

### Outcomes

The main outcomes were effectiveness at post-treatment and acceptability. Effectiveness was evaluated using a single outcome measuring OCD symptom severity in each study, determined via a hierarchy algorithm based on its frequency of usage in preceding literature (see Supplementary Appendix C). Acceptability was operationalised as the study drop-out rate for any cause during the acute treatment phase.

### Statistical analyses

A series of pairwise meta-analyses were performed for all direct comparisons using a random-effects pooling model, with the *I*^2^ statistic and *τ*^2^ calculated to assess the heterogeneity of effect sizes.

NMAs were conducted to evaluate the comparative effectiveness of all treatments by combining both direct and indirect evidence. Initially, we depicted the geometry of the network of evidence using network plots.^41^ Subsequently, NMAs were conducted for both effectiveness and acceptability utilising a frequentist NMA method,^42^ with the random-effects model.^43^ Comparative standard mean differences (SMDs) for effectiveness and relative risks for acceptability were reported with 95% confidence intervals. The ranking of treatment formats was assessed based on the surface under the cumulative ranking (SUCRA) curve.^44^ Furthermore, we examined the results at 3–12 months follow-up.

To assess the assumption of transitivity, we created a table of characteristics of patients and interventions for direct comparisons, verifying if potential effect modifiers were similarly distributed across the network. Consistency of the network was checked through both local and global inconsistency tests. The net heat plot^45^ visualised the degree of inconsistency within the network using colour gradients, as determined in the local test. The node-splitting method^46^ was employed to scrutinise consistency across the entire network of treatments, assessing the consistency assumption for each node (treatment comparison) in the network during the global test.

Three pre-planned sensitivity analyses were conducted: (a) exclusion of outliers (where the 95% CI around a study's effect size did not overlap with 95% of the pooled effect size in any pairwise meta-analyses); (b) limitation to studies with a low risk of bias; and (c) inclusion of treatment arms containing both cognitive and behavioural therapeutic techniques. In addition, a *post hoc* sensitivity analysis was performed to assess the impact of comorbidities (where studies recruited participants with at least one co-occurring mental disorder).

The analyses were conducted in R version 4.2.3 (R Foundation for Statistical Computing, Vienna, Austria; see https://www.R-project.org/) using the ‘netmeta’ package.^47^ The network plot and the certainty of evidence for the network estimates was examined through CINeMA (Confidence in Network Meta-Analysis; see https://cinema.ispm.unibe.ch/).^48^

## Results

### Identification, inclusion and characteristics of studies

**Figure 1:**
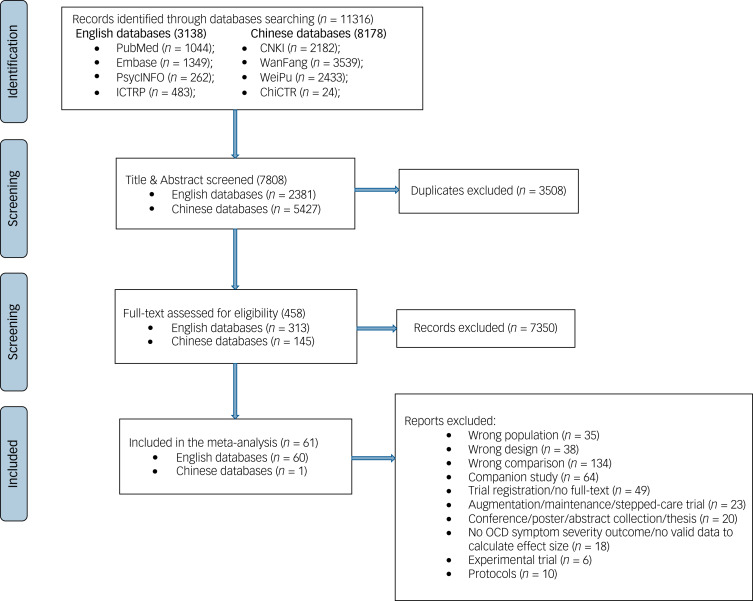
Preferred Reporting Items for Systematic reviews and Meta-Analyses flowchart of study selection and inclusion. ICTRP, international clinical trials registry platform; CNKI, China National Knowledge Infrastructure; ChiCTR, Chinese Clinical Trial Registry; OCD, obsessive–compulsive disorder.

Figure 1 presents a Preferred Reporting Items for Systematic reviews and Meta-Analyses (PRISMA) flowchart of study selection and inclusion. A total of 11 316 records were initially identified through both international and Chinese databases. Ultimately, 61 RCTs^4–22,37,49–89^ were included in the NMA, including one Chinese RCT, encompassing 3710 OCD patients (3044 adults, 666 children/adolescents). Three RCTs focused on OCD with comorbidity, including substance misuse,^53^ autism spectrum disorder^75^ and autism symptoms.^86^

Table 1 provides an overview of the characteristics of the included studies, and more detailed information can be found in Supplementary Appendices D and E.

**Table 1:** Characteristics of included studies in effectiveness and acceptability network

Characteristics	Effectiveness network		Acceptability network	
Number of studies	61		46	
Total number of patients included	3710		3035	
Adult (>18)	3044		2424	
Children/adolescent (≤18)	666		611	
Mean age (years)	6–43		7–43	
% Female (median)	57		59	
% Psychiatric medicine use (median)	48		48	
	*N*	%	*N*	%
Comorbidity with mental disorders	3	5	3	7
Publication year				
1992–2000	5	8	2	4
2001–2010	22	36	16	35
2011–2023	34	56	28	61
Region where studies were conducted				
USA	21	34	12	26
Europe	16	26	15	33
Asia	7	11	6	13
Australia	6	10	4	9
UK	6	10	6	13
Canada	2	3	1	2
Other	3	5	2	4
Recruitment way				
Clinical	22	36	16	35
Community	36	59	28	61
Other	3	5	2	4
Number of sessions				
1–7 sessions	6	10	5	11
8–15 sessions	43	70	34	74
16–27 sessions	9	15	5	11
Unclear	3	5	2	4
At post-treatment				
1–7 weeks	13	21	9	20
8–15 weeks	37	61	31	67
16–27 weeks	10	16	6	13
Unclear	1	2	–	–
At follow-up				
3–12 months	16		15	
Was a treatment protocol/manual used?				
Yes	56	92	44	96
Unclear	5	8	2	4
Was treatment fidelity checked?				
Yes	49	80	38	83
Unclear	12	20	8	17
Was treatment delivered by professionals?				
Yes	47	77	40	87
Unclear	5	8	–	
Not applicable	9	15	6	13
Outcome measure				
(C)Y-BOCS	57	93	44	96
Other	4	7	2	4
Risk of bias				
Low risk	2	3	2	4
Some concerns	16	26	14	30
High risk	43	71	30	66
Adequate sequence generation	32	52	28	61
Allocation concealment	26	43	24	52
Intent-to-treat analyses	35	57	32	70
Blinded outcome assessor	48	79	37	80
Selective outcome reporting	50	82	36	78

Y-BOCS, Yale–Brown Obsessive–Compulsive Scale; CY-BOCS, Children's Yale–Brown Obsessive–Compulsive Scale.

Within 61 RCTs, there were a total of 73 comparisons. A total of 33 treatment arms were individual, 12 were group, 11 were family involved, 11 were unguided self-help, 9 were guided self-help, 5 were remote delivery and 3 were time intensive. For the arms of control groups, 32 were waitlist, 17 were psychological placebo, 7 were care-as-usual and 3 were pill placebo.

Over half of the included RCTs were conducted within the past 15 years, and 60% were carried out in USA and Europe. Most participants (59%) were recruited from the community. Treatment typically ranged from 8 to 15 sessions. A high proportion (92%) of RCTs reported explicitly adherence to treatment protocols or manuals, while 80% monitored treatment fidelity and 77% delivered treatment through trained therapists. The Yale–Brown Obsessive–Compulsive Scale (Y-BOCS) and Children's Y-BOCS (CY-BOCS) were predominantly utilised for assessments. Post-treatment assessments were mostly conducted at 8–15 weeks.

Concerning the risk of bias (see Supplementary Appendix F), 52% of RCTs reported adequate sequence generation, 43% reported allocation to conditions by an independent third party and 79% reported self-reported or blinded clinician-rated outcomes. More than half of RCTs (57%) used adequate analyses for handling missing data. However, 82% of RCTs raised concerns regarding selective outcome reporting. In total, 71% of RCTs were rated as having a high risk of bias.

### Network plot

**Figure 2:**
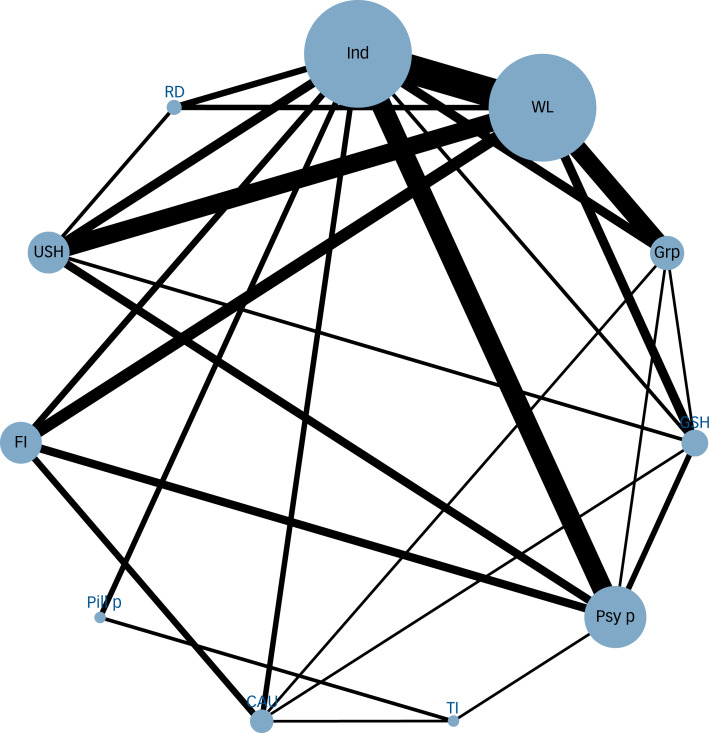
Network plot of meta-analysis. The size of node and thickness of edges were based on the number of studies on that comparison. Ind, individual; Grp, group; RD, remote-delivery; FI, family involved; GSH, guided self-help; USH, unguided self-help; TI, time intensive; WL, waitlist; pill p, pill placebo; Psy p, psychological placebo; CAU, care-as-usual.

The network plot is presented in Fig. 2. Overall, the network was well connected. Individual was the most studied CBT delivery format, demonstrating strong connections with other formats and control groups. Individual, remote-delivery, group, guided self-help, unguided self-help and family-involved formats exhibited connectivity primarily through comparisons with waitlist and psychological placebo. The time-intensive format was the least studied, with only three trials comparing it to control groups.

### Pairwise meta-analyses

The most direct comparisons were that between individual and waitlist formats. The time-intensive format had the fewest direct comparisons. Individual, remote-delivery, group, guided self-help, unguided self-help and family-involved formats were significantly more effective than waitlist format (SMD, −0.53 to −1.82). The results of pairwise meta-analyses for CBT delivery formats can be found in Supplementary Appendix G.

### Network meta-analyses

#### Effectiveness

Table 2 presents the NMA results for effectiveness and acceptability. Individual, remote-delivery, group, family-involved, guided self-help and time-intensive formats were significantly more effective than waitlist (SMD, −1.66 to −0.87), psychological placebo (SMD, −1.37 to −0.58), pill placebo (SMD, −1.57 to −0.78) and care-as-usual (SMD, −1.54 to −0.75) formats. Unguided self-help was significantly more effective than waitlist (SMD, −0.67), psychological placebo (SMD, −0.39) and care-as-usual (SMD, −0.56) formats, but significantly less effective than individual (SMD, −0.58), remote-delivery (SMD, –0.80), family-involved (SMD, −0.65) and time-intensive (SMD, −0.98) formats. In addition, individual (SMD, −0.38), remote-delivery (SMD, −0.60) and family-involved (SMD, −0.46) formats were significantly more effective than group format. No significant differences were observed among individual, remote-delivery, family-involved, guided self-help and time-intensive formats.

**Table 2:** Network meta-analyses of cognitive–behavioural therapy delivery formats for obsessive–compulsive disorder

**Individual**	0.94 (0.84; 1.04)	0.99 (0.91; 1.07)	0.96 (0.87; 1.05)	1.06 (0.78; 1.43)	0.99 (0.91; 1.07)	**1.11 (1.02; 1.20)**	1.1 (0.82; 1.47)	1.11 (0.87; 1.42)	0.99 (0.93; 1.06)	1.00 (0.95; 1.07)
0.22 (−0.27; 0.71)	**Remote delivery**	1.05 (0.93; 1.20)	1.02 (0.89; 1.17)	1.13 (0.82; 1.56)	1.06 (0.93; 1.20)	**1.18 (1.04; 1.35)**	1.17 (0.86; 1.60)	1.19 (0.91; 1.55)	1.06 (0.94; 1.20)	1.07 (0.96; 1.21)
0.08 (−0.28; 0.43)	−0.15 (−0.73; 0.44)	**Family involved**	0.97 (0.87; 1.08)	1.07 (0.79; 1.45)	1.00 (0.91; 1.10)	**1.12 (1.02; 1.24)**	1.11 (0.84; 1.48)	1.13 (0.88; 1.45)	1.01 (0.93; 1.09)	1.02 (0.95; 1.10)
**−0.38 (−0.71; −0.05)**	**−0.60 (−1.17; −0.03)**	**−0.46 (−0.89; −0.02)**	**Group**	1.11 (0.81; 1.51)	1.04 (0.94; 1.14)	**1.16 (1.04; 1.29)**	1.15 (0.86; 1.55)	1.16 (0.90; 1.51)	1.04 (0.94; 1.14)	1.05 (0.97; 1.14)
0.41 (−0.35; 1.16)	0.18 (−0.71; 1.08)	0.33 (−0.47; 1.13)	0.79 (−0.01; 1.59)	**Time intensive**	0.94 (0.69; 1.27)	1.05 (0.77; 1.43)	1.04 (0.89; 1.21)	1.05 (0.78; 1.43)	0.94 (0.69; 1.27)	1.02 (0.94; 1.09)
−0.25 (−0.63; 0.12)	−0.48 (−1.07; 0.12)	−0.33 (−0.79; 0.13)	0.13 (−0.31; 0.56)	−0.66 (−1.47; 0.15)	**Guided self-help**	**1.12 (1.02; 1.22)**	1.11 (0.83; 1.49)	1.13 (0.87; 1.45)	1.00 (0.93; 1.08)	0.95 (0.70; 1.29)
**−0.58 (−0.89; −0.26)**	**−0.80 (−1.35; −0.25)**	**−0.65 (−1.07; −0.23)**	−0.20 (−0.60; 0.21)	**−0.98 (−1.79; −0.18)**	−0.32 (−0.74; 0.10)	**Unguided self-help**	0.99 (0.74; 1.33)	1.01 (0.78; 1.30)	**0.90 (0.83; 0.97)**	**0.91 (0.84; 0.98)**
**−1.13 (−1.60; −0.67)**	**−1.36 (−2.02; −0.69)**	**−1.21 (−1.71; −0.71)**	**−0.75 (−1.27; −0.24)**	**−1.54 (−2.30; −0.79)**	**−0.88 (−1.41; −0.35)**	**−0.56 (−1.09; −0.03)**	**Care-as-usual**	1.01 (0.74; 1.38)	0.90 (0.68; 1.21)	0.91 (0.68; 1.22)
**−1.16 (−1.82; −0.50)**	**−1.38 (−2.20; −0.56)**	**−1.23 (−1.97; −0.50)**	**−0.78 (−1.51; −0.04)**	**−1.57 (−2.35; −0.78)**	**−0.90 (−1.65; −0.15)**	−0.58 (−1.31; 0.15)	0.02 (−0.74; 0.78)	**Pill placebo**	0.89 (0.69; 1.14)	0.90 (0.70; 1.16)
**−0.96 (−1.23; −0.70)**	**−1.19 (−1.73; −0.64)**	**−1.04 (−1.40; −0.68)**	**−0.58 (−0.96; −0.21)**	**−1.37 (−2.14; −0.60)**	**−0.71 (−1.09; −0.33)**	**−0.39 (−0.73; −0.04)**	0.17 (−0.32; 0.66)	0.20 (−0.51; 0.90)	**Psychological placebo**	1.01 (0.94; 1.08)
**−1.25 (−1.49; −1.01)**	**−1.47 (−1.98; −0.97)**	**−1.33 (−1.67; −0.98)**	**−0.87 (−1.18; −0.56)**	**−1.66 (−2.43; −0.88)**	**−1.00 (−1.36; −0.63)**	**−0.67 (−0.97; −0.37)**	−0.12 (−0.60; 0.37)	−0.09 (−0.79; 0.61)	−0.29 (−0.59; 0.01)	**Waitlist**

Standardised mean differences (SMDs) and 95% confidence intervals for effectiveness are reported below the diagonal. SMDs lower than 0 favour the column-defining treatment, with 95% confidence intervals not including the point of no difference (0) highlighted in bold. Relative risks and 95% confidence intervals for acceptability are reported above the diagonal. relative risks greater than 1 favour the column-defining treatment, with 95% confidence intervals not including the point of no difference (1) highlighted in bold.

#### Acceptability

No significant differences were found among individual, remote-delivery, group, guided self-help, family-involved, time-intensive and control groups. However, unguided self-help was found to be significantly less acceptable than individual (relative risk, 1.11), remote-delivery (relative risk, 1.18), group (relative risk, 1.16), guided self-help (relative risk, 1.12) and family-involved (relative risk, 1.12) formats. Moreover, unguided self-help demonstrated significantly lower acceptability (relative risk, 0.90 to 0.91) compared to psychological placebo and waitlist formats.

### Ranking of the treatment formats

**Figure 3:**
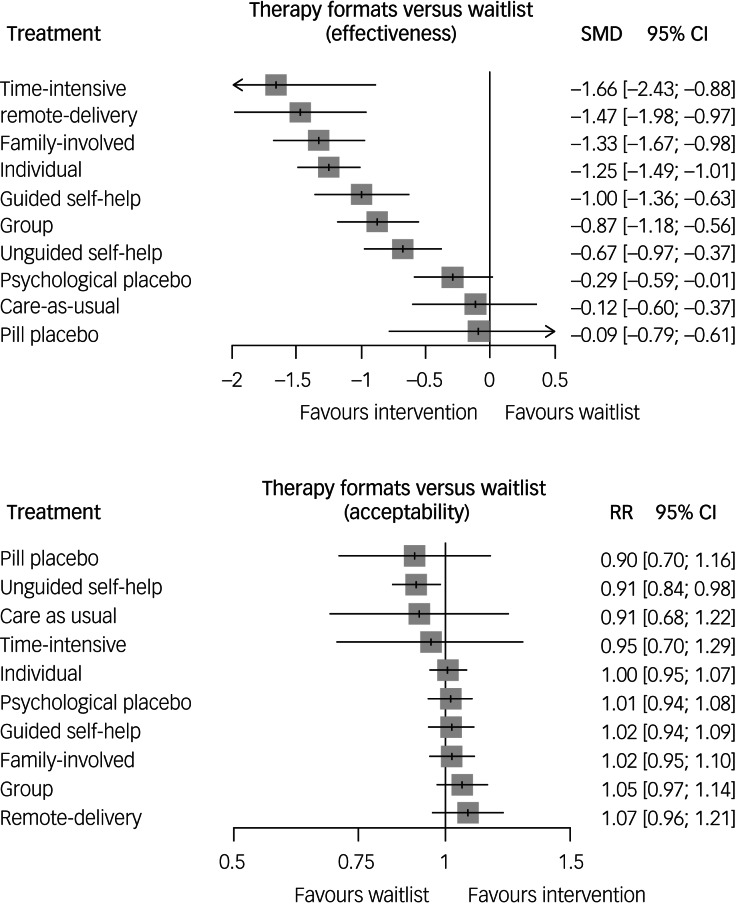
Ranked forest plot of effectiveness and acceptability of cognitive–behavioural therapy formats. SMD, standard mean difference; 95% CI, 95% confidence interval; RR, relative risk.

Figure 3 presents a forest plot illustrating the ranking of treatment formats, with waitlist as the reference group. A scatter plot combining effectiveness and acceptability is available in Supplementary Appendix H, where detailed SUCRA rankings of the treatment formats can also be found. For effectiveness, time-intensive ranked the highest (92%), followed by remote-delivery (87%), family-involved (80%), individual (76%), guided self-help (59%), group (52%) and unguided self-help (42%) formats. Regarding acceptability, remote-delivery (81%) held the highest rank, followed by family-involved (61%), guided self-help (59%), individual (54%) and time-intensive (41%) formats. Unguided self-help (15%) ranked considerably lower than other formats. In terms of confidence in the quality of evidence assessed through CINeMA, none of the comparisons were rated as ‘high confidence’, primarily because of within-study bias. Confidence in the estimate ranged from moderate to low (see Supplementary Appendix I).

### Consistency examination

Visual inspection of the distribution of potential effect modifiers indicated that most potential modifiers were similarly distributed across the comparisons in the network, although there were potential differences in mean age and gender across some comparisons (see Supplementary Appendix J). However, our previous study found that age and gender were not effect modifiers.^33^ Both local and global tests generally demonstrated substantial consistency between direct and indirect evidence, except in some comparisons with a limited number of studies, such as time intensive versus psychological placebo (see Supplementary Appendix K).

### Sensitivity analyses

Sensitivity analyses excluding outliers^62^ and studies with comorbidities^53,75,86^ yielded results consistent with the main analyses (see Supplementary Appendices L and M). In the sensitivity analysis for studies with low risk of bias, 20 comparisons were included (see Supplementary Appendix N). The results were generally similar, with minor differences compared to the main analyses. All CBT delivery formats were more effective than care-as-usual. No significant differences were found among most formats, except for unguided self-help, which was less effective than individual and remote-delivery formats. In the sensitivity analysis for treatment arms containing both cognitive and behavioural therapeutic techniques, 40 comparisons were included (see Supplementary Appendix O). No significant differences were found among all formats. They were significantly more effective than waitlist, with the exception of the time-intensive format, which exhibited no significant difference in effect compared to waitlist.

### Long-term effect

Sixteen direct comparisons examining the effects of CBT delivery formats at 3–12 months follow-up were identified, with 12 focusing on individual and the remaining four on group, guided self-help and unguided self-help formats. A NMA was performed to estimate the long-term effects of CBT delivery formats, using psychological placebo as the reference group. Individual CBT continued to be effective at 3–12 months follow-up compared to psychological placebo (SMD, −0.46) and unguided self-help formats (SMD, −0.72; see Supplementary Appendix P).

## Discussion

This NMA represents the first comprehensive comparison of the effectiveness and acceptability of various CBT delivery formats for OCD in both paediatric and adult populations. The results indicated that all CBT delivery formats significantly reduced OCD symptom severity compared to control groups, and these findings were consistent across all sensitivity analyses. Moreover, most CBT delivery formats showed no significant difference in reducing OCD symptom severity, such as individual CBT versus remote-delivery CBT and individual CBT versus guided self-help CBT. This is partly consistent with a previous NMA on paediatric OCD^31^ that included 20 comparisons for in-person CBT, 4 for telephone/webcam CBT and 2 for guided self-help CBT, finding no significant difference between individual and telephone/webcam CBT, but noting that individual CBT was more effective than guided self-help CBT. Our findings underscore the potential of various CBT delivery formats as viable alternatives to traditional individual, face-to-face weekly CBT. However, further research with direct comparisons among delivery formats, especially those with limited current evidence such as time-intensive formats, is needed for more definitive conclusions.

However, group CBT was less effective than individual CBT, which contrasts with findings from previous conventional meta-analyses directly comparing these formats.^28,29^ In addition, group CBT was less effective than remote-delivery and family-involved CBT. Despite this, group CBT showed great advantages in terms of acceptability, supporting the hypothesis that it might prevent drop out, as participants tend to increase their motivation to engage in treatment after sharing feelings with others facing similar conditions, particularly for less motivated patients with weaker symptom insight.^90^ These results should be interpreted with caution because of the small number of comparisons included and the inconsistent results from sensitivity analyses.

Furthermore, while unguided self-help showed less effectiveness compared to other treatment formats, the effects of this format were still moderate. Similar findings have been observed for unguided interventions in depression.^91^ Nonetheless, unguided self-help has the advantage of reaching individuals with OCD who face barriers to accessing traditional treatments, such as geographical isolation, a shortage of trained therapists, treatment costs and stigma.^92–95^ Previous research has indicated that unguided self-help has the potential to reduce stigma associated with seeking professional help,^96^ indicating its potential for significant clinical impact despite its high drop-out rates. The acceptability results for unguided self-help corroborate the results of a prior study,^33^ wherein unguided self-help interventions displayed a significantly higher drop-out rate compared to control groups, and the study further highlighted the association among the type of treatment, recruitment method of participants and drop-out rates. Future studies on predictors and moderators of drop out in CBT intervention at the patient level could be helpful for enhancing treatment adherence.

Regarding treatment rank, time-intensive format displayed a high potential for reducing OCD symptomology. However, the limited number of studies (*n* = 3) necessitates a cautious interpretation of the results. The remote-delivery format showed promise in enhancing the accessibility of CBT, combining effectiveness and acceptability. Remote-delivery CBT provides psychotherapeutic skills and treatment sessions similar to traditional face-to-face CBT but utilises technology such as telephone or videoconferencing to facilitate real-time interactions between therapists and clients who are physically apart. This format is particularly beneficial for individuals who cannot access traditional CBT because of geographical or social barriers, or pandemic-related restrictions. The potential for improved treatment efficacy with remote-delivery CBT is noted, as clients receive therapeutic support in real time. These findings align with the National Institute for Health and Clinical Excellence guidelines, which support telephone-delivered CBT.^97^ Family-involved CBT also showed notable effectiveness and acceptability; in these interventions, family members are recognised to play a significant role in either exacerbating or maintaining the OCD symptoms of patients through overly accommodating or overly antagonising behaviors.^34^ Common treatment components for family members in these interventions include psychoeducation on OCD, strategies to reduce involvement in patients’ symptoms and encouragement of family support for home-based treatment exercises.^18,50,78^ It is plausible that patients with positive influences from their families may have increased confidence in adhering to treatment and practising exercises in their natural environments. While a thorough exploration of the moderators of effect in family interventions is highly necessary, these results underscore the importance of incorporating family members in future clinical practices for OCD treatment. In addition, guided self-help also exhibited advantages in terms of both effectiveness and acceptability, suggesting its significant potential in treating OCD on a larger scale.^98^ However, given that most CBT delivery formats did not differ significantly in terms of effectiveness and acceptability, treatment rankings should be viewed as a general guidance rather than definitive conclusions. In selecting a treatment, additional factors such as cost-effectiveness are also crucial. For instance, while telephone-delivered CBT showed no significant difference in cost-effectiveness compared to individual CBT,^99^ guided self-help formats, such as internet-delivered CBT, were demonstrated to be cost-effective options.^100^ Ultimately, the choice of treatment should be based on individual needs when multiple evidence-based options are available.

In terms of long-term effects, the limited number of studies examining the lasting effects of CBT may be attributed to the nature of comparisons included. Given that most control groups involved waitlist and psychological placebo conditions, follow-up data often became unusable owing to participants receiving the intervention after the post-test. Consistent with the prior research,^101^ individual CBT remained more effective than the control group at 3–12 months follow-up. However, caution is warranted when interpreting results for other CBT formats because of the limited number of studies included in this NMA.

### Limitations

Several limitations should be noted in this NMA. First, studies were unevenly distributed among different delivery formats: while most studies focused on individual CBT, few studies focused on time-intensive CBT. This imbalance affects the validity of the findings and necessitates cautious interpretation of the results. Second, high risk of bias observed across studies affected the confidence in the results. Future studies should employ more rigorous and stringent methodologies, especially in handling missing data and preventing selective reporting of outcomes through prospective registration. Third, the validity of transitivity assumption is not fully assured, and further research is needed to explore differences among groups with OCD, such as those using guided self-help, time-intensive or family-involved treatments. Fourth, more future studies are required to better understand long-term effects. Lastly, we used drop-out rates as a proxy for acceptability, acknowledging that it may not fully capture the complexity of patients’ experiences and decisions to continue or discontinue treatment. Future research should aim to incorporate more nuanced and direct measures of acceptability.

In conclusion, most CBT delivery formats emerge as potential alternatives to conventional individual CBT; delivering remotely or involving family members may offer advantages for both treatment effect and adherence. Intensive treatment delivery reveals strong potential for reducing OCD symptomology, but more robust evidence is required because of a limited number of studies. Although displaying less effectiveness than most other formats, unguided interventions hold significant potential to extend the accessibility of treatments, particularly in environments with limited resources. More research on direct comparisons between formats is highly recommended. Ensuring the application of effective and acceptable delivery formats across diverse settings and populations is crucial for OCD management. The findings from this study contribute valuable insights into the optimisation and organisation of mental health resources that can shape future global clinical guidelines for managing OCD.

## Supporting information

Wang et al. supplementary material Wang et al. supplementary material

## Data Availability

The data that support the findings of this study are available from the corresponding author Y.W., upon reasonable request.
