# Effectiveness and acceptability of cognitive–behavioural therapy delivery formats for obsessive–compulsive disorder: network meta-analysis – CORRIGENDUM

**DOI:** 10.1192/bjp.2025.10527

**Published:** 2026-03

**Authors:** Yingying Wang, Clara Miguel, Marketa Ciharova, Arpana Amarnath, Jingyuan Lin, Ruiying Zhao, Marieke B. J. Toffolo, Mathias Harrer, Sascha Y. Struijs, Leonore M. de Wit, Pim Cuijpers

This article originally published with an error in the legend of Table 2. To avoid potential misinterpretation of information, the error has been corrected in all versions of the article.
