# Supplementary material for: Effectiveness and acceptability of cognitive–behavioural therapy delivery formats for obsessive–compulsive disorder: network meta-analysis
Source: Br J Psychiatry. 2026 Mar;228(3):252–62. doi: 10.1192/bjp.2024.197 (PMC12912871; doi:10.1192/bjp.2024.197)
Supplement: Wang et al. supplementary material [file S0007125024001971sup001.docx]

Appendix A. Search Strings in the international and Chinese databases

Appendix B. The definitions of different CBT delivery formats

Appendix C. Hierarchy of OCD symptom severity measurements

Appendix D. Key characteristics of included studies

Appendix E. Key characteristics of interventions

Appendix F. Assessment of risk of bias

Appendix G. Pairwise meta-analyses of CBT delivery formats

Appendix H. Rank of CBT delivery formats by SUCRA

Appendix I. GRADE appraisal (CINeMA)

Appendix J. Transitivity assessment: characteristics of included studies across different comparisons

Appendix K. Inconsistency check – local and global test

Appendix L. Sensitivity analysis: Outlier excluded

Appendix M. Sensitivity analysis: Studies with comorbidity excluded

Appendix N. Sensitivity analysis: Only studies with low risk of bias

Appendix O. Sensitivity analysis: CBT studies combining both cognitive and behavioural therapeutic techniques

Appendix P. Long-term effects at 3 – 12 month

**Appendix A. Search Strings in the international and Chinese databases**

**International databases**

**PubMed search**

("obsessive compulsive"[Title/Abstract] OR "OCD"[Title/Abstract]) AND (randomizedcontrolledtrial[Filter])

**Embase**

'obsessive compulsive':ti,ab,kw OR ocd:ti,ab,kw

AND 'randomized controlled trial'/de

**PsycINFO**

MA "obsessive compulsive" or OCD 
Expanders - Apply equivalent subjects

Narrow by Methodology: - clinical trial

Search modes - Boolean/Phrase

**WHO’s Registry (international clinical trials registry platform of WHO)**

“obsessive compulsive” or OCD

**Chinese databases**

**CNKI (TKA=Title/Keyword/Abstract; FT=Full Text)**

TKA = ('强迫症' + '强迫性神经官能症' + '强迫性障碍' + '强迫障碍' + '强迫性神经症') AND TKA = ('治疗' + '疗法' + '干预' + '医治' + '辅导') AND (FT= ('临床试验'+'随机对照试验') OR TKA= ('临床'+'试验'+'随机'+'交叉'+'组'+'安慰剂'+'双盲'+'单盲'+'三盲'+'平行组'+'对照研究'+'对照试验'+'对照设计'+'随机对照'+'随机对照研究'+'随机对照临床试验'+'随机对照临床研究'+'随机对照实验'+'随机化'+'RCT'))

同义词扩展

**WanFang (题名或关键词=Title or Keyword; 摘要=Abstract)**

((题名或关键词:(强迫症 or 强迫性神经官能症 or 强迫性障碍 or 强迫障碍 or 强迫性神经症) AND 题名或关键词:(治疗 or 疗法 or 干预 or 医治 or 辅导)) or (摘要:(强迫症 or 强迫性神经官能症 or 强迫性障碍 or 强迫障碍 or 强迫性神经症) AND 摘要:(治疗 or 疗法 or 干预 or 医治 or 辅导)))

AND (全部: (临床试验 OR 随机对照试验) OR 题名或关键词: (临床 OR 试验 OR 随机 OR交叉 OR 组 OR 安慰剂OR 双盲OR 单盲OR 三盲OR 平行组 OR 对照研究 OR 对照试验 OR 对照设计 OR 随机对照 OR 随机对照研究 OR 随机对照临床试验 OR 随机对照临床研究 OR 随机对照实验 OR 随机化 OR RCT) OR 摘要: (临床 OR 试验 OR 随机 OR交叉 OR 组 OR 安慰剂OR 双盲OR 单盲OR 三盲OR 平行组 OR 对照研究 OR 对照试验 OR 对照设计 OR 随机对照 OR 随机对照研究 OR 随机对照临床试验 OR 随机对照临床研究 OR 随机对照实验 OR 随机化 OR RCT))

主题词扩展

**WeiPu (M=Title/Keyword; R=Abstract)**

((M=(强迫症+强迫性神经官能症+强迫性障碍+强迫障碍+强迫性神经症) AND M=(治疗+疗法+干预+医治+辅导)) OR (R=(强迫症+强迫性神经官能症+强迫性障碍+强迫障碍+强迫性神经症) AND R=(治疗+疗法+干预+医治+辅导)))

AND (U=(临床试验 OR 随机对照试验) OR (M=(临床 OR 试验 OR 随机 OR交叉 OR 组 OR 安慰剂OR 双盲OR 单盲OR 三盲OR 平行组 OR 对照研究 OR 对照试验 OR 对照设计 OR 随机对照 OR 随机对照研究 OR 随机对照临床试验 OR 随机对照临床研究 OR 随机对照实验 OR 随机化 OR RCT)) OR (R=(临床 OR 试验 OR 随机 OR交叉 OR 组 OR 安慰剂OR 双盲OR 单盲OR 三盲OR 平行组 OR 对照研究 OR 对照试验 OR 对照设计 OR 随机对照 OR 随机对照研究 OR 随机对照临床试验 OR 随机对照临床研究 OR 随机对照实验 OR 随机化 OR RCT)))

**ChiCTR(Chinese Clinical Trial Registry)**

强迫

**Appendix B. The definitions of different CBT delivery formats**

| **CBT delivery format** | **Definition** |
| --- | --- |
| Individual | The treatment is delivered by the therapist in a weekly face-to-face individual setting |
| Group | The treatment is delivered by the therapist in a face-to-face group setting |
| Guided self-help | A treatment in which a professional therapist is involved in the treatment process, offering guidance to the patient using the self-help materials (administered through the internet, telephone or other media, such as a book). |
| Unguided self-help | A treatment in which individuals work independently on the program/treatment protocol to learn and apply psychotherapeutic strategies. It could be delivered in text, audio, or video format via various ways such as a book, Internet, a mobile phone application, or a website |
| Family-involved | A treatment in which family members are involved in the treatment for patients. Family may be integrated into treatment in a variety of ways: 1) join the patient in treatment 2) receive treatment independent of the patient (alone or in a group with other family members 3) a combination of the two. |
| Time-intensive | A treatment defined by a maximum total duration of 4 weeks, requiring a minimum of 10 therapist hours overall, with an average weekly therapist commitment of at least 5 hours. |
| Remote-delivery | In remote-delivery CBT, the skills and session length are typically equivalent to those in traditional face-to-face CBT. However, the sessions are conducted using technological aids, allowing real-time interaction between the patient and therapist. Common technologies used include traditional or internet-based videoconferencing and telephone. |

**The differences between this review and its registered protocol are:**

1) We used the frequentist network meta-analysis method instead of the contrast-based network meta-analysis method.

2) We set the hierarchy of OCD symptom severity measurements based on its frequency of usage in proceeding literature (we did not set the hierarchy of OCD symptom severity measurements in the protocol)

3) Pos-doc analyses were conducted for the addition of remote-delivery format as a new node, and a further sensitivity analysis focusing on studies without comorbidity.

**Appendix C. Hierarchy of OCD symptom severity measurements – based on its frequency of usage in proceeding literature**

| **Hierarchy** | **Symptom severity rating scales** | | Abbreviations |
| --- | --- | --- | --- |
| 1 | Yale-Brown Obsessive-Compulsive scale* | Clinician-rated | Y-BOCS |
| 2 | Yale-Brown Obsessive-Compulsive scale | Self-rated | Y-BOCS |
| 3 | Obsessive-Compulsive Inventory (-Revised) | Self-rated | OCI (-R) |
| 4 | Dimensional Obsessive-Compulsive Scale (Short-Form) | Self-rated | DOCS (-SF) |
| 5 | National Institute of Mental Health Obsessive-Compulsive Scale | Clinician-rated | NIMHOC |
| 6 | National Institute of Mental Health Global Obsessive Compulsive Scale | Clinician-rated | NIMHGOCS |
| 7 | The Padua Inventory | Self-rated | PAUDA |
| 8 | Maudsley Obsessional-Compulsive Inventory | Self-rated | MOCI |
| 9 | Vancouver Obsessional Compulsive Inventory | Self-rated | VOCI |
| 10 | Dimensional-YBOCS | Clinician-rated | D-YBOCS |

*Including children’s version, CY-BOCS

**Appendix D. Key characteristics of included studies**

Abbreviations: ind, individual; grp, group; gsh, guided self-help; ush, unguided self-help; FI, family-involved; TI, time-intensive; RD, remote-delivery; wl, waitlist; cau, care-as-usual; psy placebo, psychological placebo; % Med_treat: proportion of participants using psychiatric medicine in the treatment group; % Med_Ctr, proportion of participants using psychiatric in the control group; Comorbid, comorbidity of mental disorder (when participants exhibiting at least one shared type of mental disorder); US, United States; UK, United Kingdom; Recru, recruitment method of participants (com, community; clin, clinical setting; oth, others including unclear situation); Sample, the analysed sample (co, completers analysis; itt, intention-to-treat analysis); Y-BOCS, the Yale-Brown Obsessive-Compulsive Scale; CY-BOCS, the Children’s Yale-Brown Obsessive Compulsive Scale; NIMHOC, the National Institute of Mental Health Obsessive-Compulsive Scale; DOCS, the Dimensional Obsessive-Compulsive Scale; VOCI, the Vancouver Obsessional Compulsive Inventory

**Appendix E. Key characteristics of interventions**

| **Unique ID** | **First author, year** | **Name of intervention** | **Control group** | **Manual reference / articles / books upon which manual was based** | | **Were intervention delivered by a licensed psychologist / psychiatrist / psychology Master or PhD / other professionals?** | | **Was treatment integrity verified?** |
| --- | --- | --- | --- | --- | --- | --- | --- | --- |
| 1 | Alcolado,2016 | Individual cognitive therapy | Waitlist | Yes | The intervention was a manualized cognitive-behavioural module developed by the authors for the purpose of the current study, in collaboration with their research team, and in consultation with other experts in the cognitive-behavioural treatment for OCD. | NR |  | Yes |
| 2 | Anderson,2007 | Individual CBT | Waitlist | Yes | Rees, C., & Nathan, P. (2001). Obsessive Compulsive Disorder Group Treatment Program: A group cognitive behavioural programme. Nedlands, Western Australia: Riobay Enterprises. | Yes | trained postgraduate level clinical psychology students | Yes |
|  |  | Group CBT |  |  |  |  |  |  |
| 3 | Andersson,2012 | Guided self-help CBT | Psychologcial placebo (supportive therapy) | Yes | Abramowitz JS (2006). The psychological treatment of obsessive-compulsive disorder. Canadian Journal of Psychiatry 51, 407–416. Abramowitz JS (2009). Getting over OCD: a 10-step Workbook for Taking Back Your Life. Guilford Press : New York. | Yes | all clinical psychology students in their final year of the 5-year psychology programme | Yes |
| 4 | Barrett,2003 | Family-involved CBT | Waitlist | Yes | March JS, Mulle K, Herbel B (1994). Behavioral psychotherapy for children and adolescents with obsessive-compulsive disorder: An open trial of a new protocol-driven treatment package. Journal of the American Academy of Child and Adolescent Psychiatry, 33, 333-34. March JS, Mulle K (1998). OCD in children and adolescents: A cognitive-behavioral treatment manual. New York: Guilford Press. | NR |  | NR |
| 5 | Bolton,2008 | Individual ERP | Waitlist | Yes | Rachman, S. J., & Hodgson, R. (1980). Obsessions and compulsions. Englewood Cliffs, New York: Prentice-Hall. Piacentini, J., Gitow, A., Jaffer, M., Graae, M. D., & Whitaker, M. D. (1994). Outpatient behavioral treatment of child and adolescent obsessive compulsive disorder. Journal of Anxiety Disorders, 8, 277–289. | Yes | The authors, both clinicians with extensive training and experience in E/RP and CBT for OCD in children and adolescents, delivered the treatment as described above. | Yes |
| 6 | Braga,2016 | Group CBT | Waitlist | Yes | Cordioli AV, Heldt E, Braga DT, et al. Cognitive-behavioral group therapy in obsessive-compulsive disorder: a randomized clinical trial. Psychother Psychosom 2003;72:211–216. Cordioli AV, Heldt E, Bochi DB, et al. Time-limited cognitivebehavioral group therapy in the treatment ofobsessive-compulsive disorder: an open clinical trial. Rev Bras Psiquiatr 2002;24:113– 120. | Yes | All sessions were conducted by the same trained Ph.D. and masters level clinicians and assisted by an equally experienced cotherapist. All clinicians specialized in CBT for OCD, with at least 5 years ofclinical experience. | NR |
| 7 | Challacombe,2017 | Time-intensive CBT | Care-as-usual | NR |  | Yes | iCBT was predominantly delivered by the first author (F.L.C.) who is a qualified clinician, who received ongoing supervision in CBT for OCD for the duration of the study. | Yes |
| 8 | Cordioli,2003 | Group CBT | Waitlist | Yes | Cordioli AV, Heldt E, Bochi DB, Margis M, de Sousa MB, Tonello JF, Teruchkin B, Kapczinski F: Time-limited cognitive-behavioral group therapy in the treatment of obsessive-compulsive disorder: An open clinical trial. Rev Bras Psiquiatr 2002;24:113–120. | Yes | All sessions were conducted by the same therapist, assisted by a co-therapist, both specialized in psychiatry and with a CBT experience of at least 10 years. | NR |
| 9 | Fals-Stewart,1992 | Individual ERP | Psychologcial placebo (progressive muscle relaxation) | NR |  | Yes | behavior therapists with expertise in treating OCD. | NR |
| 10 | Foa,2005 | Time-intensive ERP | Pill placebo | Yes | Kozak MJ, Foa EB: Mastery of Obsessive-Compulsive Disorder: A Cognitive Behavioral Approach. San Antonio, Tex, Graywind Publications, 1997 | Yes | Exposure and ritual prevention therapists received training and ongoing weekly supervision from faculty from the Philadelphia site | Yes |
| 11 | Freeman,2014 | Family-involved CBT | Psychologcial placebo (relaxation training) | Yes | Choate-Summers M, Freeman J, Garcia A, Coyne L, Przeworski A, Leonard HL. Clinical considerations when tailoring cognitive behavioral treatment for young children with obsessive compulsive disorder. Educ Treat Child. In press. | Yes | Treatment providers for FB-CBT and FB-RT were clinical psychologists and clinical psychology trainees already familiarwithCBT | Yes |
| 12 | Freeman,2008 | Family-involved CBT | Psychologcial placebo (relaxation training) | Yes | Choate-Summers M, Freeman J, Garcia A, Coyne L, Przeworski A, Leonard HL. Clinical considerations when tailoring cognitive behavioral treatment for young children with obsessive compulsive disorder. Educ Treat Child. In press. | Yes | All study therapists were clinical psychology interns, postdoctoral fellows, and clinical psychologists with expertise in the application of behavior therapy with anxiety disorders, parent behavior management training, and relaxation and family-based treatment. | Yes |
| 13 | Freeston,1997 | Individual CBT | Waitlist | Yes | An updated English version based on the treatment manual is available from Mark H. Freeston. | Yes | There were four therapists, all graduate students trained in cognitive behavior therapy techniques. | Yes |
| 14 | Gomes,2016 | Family-involved CBT | Waitlist | Yes | Cordioli, A.V., Heldt, E., Bochi, D.B., Margis, M., de Sousa, M.B., Tonello, J.F., Teruchkin, B., Kapczinski, F., 2002. Cognitive-behavioral group therapy in obsessive-compulsive disorder: a clinical trial. Rev. Bras. Psiquiatr. 24, 113–120. Cordioli, A.V., Heldt, E., Braga Bochi, D., Margis, R., Basso de Sousa, M., Fonseca Tonello, J., Gus Manfro, G., Kapczinski, F., 2003. Cognitive-behavioral group therapy in obsessive-compulsive disorder: a randomized clinical trial. Psychother. Psychosom. 72, 211–216. | Yes | CBGT was delivered in 12 sessions, lasting for 2 h each, over 3 months, coordinated by two therapists with prior experience in CBGT. | NR |
| 15 | Greist,2002 | Individual ERP | Psychologcial placebo (systematic relaxation) | Yes | Marks IM, Baer L, Greist JH, et al. Home self-assessment of obssivecompulsive disorder. Use of a manual and a computer-conducted telephone interview: two US-UK studies. Br J Psychiatry 1998;172:406–412 | Yes | Behavior therapist | Yes |
|  |  | Unguided self-help ERP |  |  |  |  |  |  |
| 16 | Grunes,2001 | Family-involved ERP |  | Yes | Yaryura-Tobias, J. A., & Neziroglu, F. (1997). Bio-behavioral treatment of obsessive compulsive spectrum disorders. New York: W. W. Norton & Company. | Yes | The therapists who saw the patients were doctoral- and master's-level psychologists trained at the Institute for Bio-Behavioral Therapy and Research and experienced in the techniques of in vivo and imaginal ERP | Yes |
|  |  | Individual ERP |  |  |  |  |  |  |
| 17 | Herbst,2014 | Guided self-help ERP | Waitlist | Yes | Lakatos A, Reinecker H: Kognitive Verhaltenstherapie bei Zwangsstörungen: ein Therapiemanual, revised ed 3. Göttingen, Hogrefe, 2007. | Yes | The treatment was conducted by 3 experienced cognitive behavioral therapists, each with at least 4 years of therapeutic expertise. | NR |
| 18 | Hauschildt,2016 | Unguided self-help third-wave CBT (myMCT) | Psychologcial placebo (psychoeducation) | Yes | Moritz, S., Andreou, C., Schneider, B. C., Wittekind, C. E., Menon, M., Balzan, R. P., & Woodward, T. S. (2014). Sowing the seeds of doubt: a narrative review on metacognitive training in schizophrenia. Clinical Psychology Review, 34, 358–366 | NR |  |  |
| 19 | Jaurrieta,2008 | Individual CBT | Waitlist | Yes | McGinn, L. K., & Sanderson, W. C. (1999). Treatment ofobsessivecompulsive disorder. London: Jason Aronson. | Yes | Individual and group treatment conditions were applied and evaluated (Y-BOCS and HAM) by the same therapist, a qualified psychologist with master’s degree and 3 years of experience in OCD. | NR |
|  |  | Group CBT |  |  |  |  |  |  |
| 20 | Jónsson,2011 | Individual CBT |  | Yes | Salkovskis PM. Obsessional-compulsive problems: a cognitive-behavioural analysis. Behav Res Ther 1985;23:571–583. | Yes | all trained CBT therapists with at least 1 year of clinical experience in CBT for OCD | Yes |
|  |  | Group CBT |  |  |  |  |  |  |
| 21 | Khodarahimi,2009 | Individual ERP | Waitlist | Yes | Salkovskis, P. M., & Kirk, J. (1989). Obsessional disorders. In K. Hawton, et al. (Eds.), Cognitive behavior therapy for psychiatric problems: A practical guide. Oxford: Oxford university press. | Yes | clinical psychologist | Yes |
| 22 | Kobayashi,2020 | Family-involved ERP | Care-as-usual | Yes | Ministry of Health, Labour, and Welfare. Cognitive behavioral therapy for Obsessive-Compulsive Disorder. In: Treatment manual for therapist. Japan (2015). https://www.mhlw.go.jp/stf/seisakunitsuite/bunya/hukushi_kaigo/ shougaishahukushi/kokoro/index.html | Yes | Four clinical psychologists with doctorate or master’s degrees facilitated the FERP program. | Yes |
| 23 | Kyrios,2018 | Guided self-help CBT | Psychologcial placebo (progressive relaxation) | Yes | Kyrios M, Nedeljkovic M, Moulding R, Klein B, Austin D, Meyer D, et al. Study protocol for a randomised controlled trial of internet-based cognitive-behavioural therapy for obsessive-compulsive disorder. BMC Psychiatry 2014 Jul 25;14:209 [FREE Full text] [doi: 10.1186/1471-244X-14-209] [Medline: 25062747] | Yes | Therapists (n=10) were either psychologists or students undertaking a masters or professional doctorate in clinical psychology and underwent an online training module for e-therapists working within the Mental Health Online platform [49]. | Yes |
| 24 | Katz,2023 | Individual CBT | Waitlist | Yes | Kozak, M., & Foa, E. B. (1997). Mastery of obsessive-compulsive disorder: A cognitivebehavioral approach. The Psychological Corporation,. Clark, D. A. (2004). Cognitive-Behavioral Therapy for OCD. Guilford Press,. | Yes | All therapists were licensed clinical psychologists or psychologists under supervised practice working in specialty, university-affiliated adult assessment and treatment centres with a specialization in OCD. | Yes |
| 25 | Lenhard,2017 | Family-involved CBT | Waitlist | Yes | Lenhard F, Vigerland S, Andersson E, et al. Internet-delivered cognitive behavior therapy for adolescents with obsessive-compulsive disorder: an open trial. PLoS One. 2014;9:e100773. | Yes | Treating clinicians were 6 trained psychologists with experience in treating pediatric OCD and ICBT | Yes |
| 26 | Lewin,2014 | Family-involved ERP | Care-as-usual | Yes | Freeman, J. B., & Garcia, A. M. (2009). Family-based treatment for young children with OCD workbook. New York, NY: Oxford University Press. | Yes | Therapists were licensed clinical psychologists or advanced clinical psychology doctoral students (who had3 years’ experience in E/RP for OCD under the supervision of the principal investigator); | Yes |
| 27 | Lindsay,1997 | Time-intensive ERP | Psychologcial placebo (anxiety management traning) | Yes | Andr.ws, G.,Crlno, R., Hunt,C., at at(I994) TheTreatment ofAnxietyDisorders.NewYork:CambridgeUniversity Press | Yes | Clinicians | NR |
| 28 | Lundstrom, 2022 | Individual CBT |  | Yes | Foa EB, Yadin E, Lichner TK. Exposure and Response (Ritual) Prevention for Obsessive Compulsive Disorder: Therapist Guide. Oxford University Press; 2012. doi:10.1093/med:psych/9780195335286.001.0001. Foa EB, Yadin E, Lichner TK. Exposure and Response (Ritual) Prevention for Obsessive Compulsive Disorder: Therapist Guide. Oxford University Press; 2012. doi:10.1093/med:psych/9780195335286.001.0001. Andersson E, Steneby S, Karlsson K, et al. Long-term efficacy of Internet-based cognitive behavior therapy for obsessive-compulsive disorder with or without booster: a randomized controlled trial. Psychol Med. 2014;44(13): 2877-2887. | Yes | Therapists were 8 licensed clinical psychologists (including L.L. and O.F.), with expertise in treating OCD both face-to-face and digitally. They received supervision from the lead author (L.L.) on request and every second week at the clinic’s own scheduled supervision hours | Yes |
|  |  | Guided self-help CBT |  |  |  |  |  |  |
|  |  | Unguided self-help CBT |  |  |  |  |  |  |
| 29 | Lee,2023 | Group third-wave CBT | Waitlist | Yes | Twohig, M. (2009). The application of acceptance and commitment therapy to obsessivecompulsive disorder. Cognitive and Behavioral Practice, 16(1), 18–28. https://doi.org/ 10.1016/j.cbpra.2008.02.008 | Yes | Two psychiatrists and one clinical psychologist participated as therapists | Yes |
| 30 | Matsumoto,2022 | Guided self-help CBT | Waitlist | Yes | The ICBT program, was developed by the first author (KM) on the elearning platform (LearningBox®) of Tatsuno System Inc. | Yes | Two clinical psychologists with a PhD (one male [KM] and female [SH] in their early thirties) who completed the training in CBT and had experience providing CBT for OCD guided the participants using Share Medical | Yes |
| 31 | Mahoney,2014 | Unguided self-help CBT | Waitlist | Yes | Andrews, G., Creamer, M., Crino, R., Hunt, C., Lampe, L., & Page, A. (2003). The treatment of anxiety disorders: Clinician guides and patient manuals (2nd ed.). New York: Cambridge University Press. Bennett-Levy, J., Bulter, G., Fennell, M., Hackman, A., Mueller, M., & Westbrook, D. (2004). Oxford guide to behavioural experiments in cognitive therapy. New York: Oxford University Press. Foa, E. B. (2010). Cognitive behavioural therapy for obsessive-compulsive disorder. Dialogues in Clinical Neuroscience, 12,199e207. St Clare, T., Menzies, R. G., & Jones, M. K. (2008). Danger ideation reduction therapy (DIRT) for obsessive compulsive washers: A comprehensive guide to treatment. Bowen Hills: Australian Academic Press. | Not applicable | | Yes |
| 32 | Moritz,2018 | Unguided self-help third-wave CBT (myMCT) | Waitlist | Yes | Moritz, S., & Hauschildt, M. (2016). Metacognitive training for obsessive-compulsive disoder (myMCT). A self-help book (3rd ed.). Hamburg: VanHam Campus Press. Moritz, S., & Hauschildt, M. (2011). Erfolgreich gegen Zwangsstörungen: Metakognitives Training - Denkfallen erkennen und entschärfen [Successful against OCD. Metacognitive training - detecting and defusing cognitive traps]. Heidelberg: Springer | Not applicable | | Yes |
| 33 | Moritz,2011 | Unguided self-help CT | Waitlist | Yes | Moritz S, Jelinek L. Association splitting—self-help guide for reducing obsessive thoughts. Hamburg: VanHam Campus Verlag; 2007. Moritz S, Jelinek L, Klinge R, Naber D. Fight fire with fireflies! Association splitting: a novel cognitive technique to reduce obsessive thoughts. Behav Cogn Psychother 2007;35:631–635. | Not applicable | | Yes |
| 34 | Moritz,2010 | Unguided self-help third-wave CBT (myMCT) | Waitlist | Yes | Moritz S. Metacognitive Training for Obsessive-Compulsive Disorder (MyMCT). A Self-Help Book. [Erfolgreich gegen Zwangsstörungen. Metakognitives Training. Denkfallen erkennen und entschärfen.] Heidelberg, Germany: Springer. 2010. | Not applicable | | Yes |
| 35 | McLean,2001 | Group CBT | Waitlist | Yes | VanNoppen, B., Steketee, G., & Pato, M. (1994). Group Behavior Therapy (GET) Treatment Manual for Obsessive Compulsive Disorder (OCD). Unpublished manual. van Oppen, P.. & Arnt/., A. (1994). Cognitive therapy for obsessivecompulsive disorder. Behaviour Research and Therup\, 32, 79-87. Salkovskis, P. M. (1996). Cognitive-behavioral approaches to the understanding of obsessional problems. In R. M. Rapee (Ed.), Current controversies in the anxiety disorders (pp. 33-50). New York: Guilford Press. | Yes | The lead therapists for this study were licensed clinical psychologists, all with experience in cognitive-behavioural treatment of anxiety disorders, including OCD. Co-therapists were licensed clinical psychologists or psychology internship | Yes |
| 36 | Nakatani,2005 | Unguided self-help ERP | Psychologcial placebo (relaxation training) | Yes | Iikura Y: Treatment guide for obsessive-compulsive disorder. Osaka, Nihei-Sha, 1999. | Not applicable | | Yes |
| 37 | Norman,2021 | Individual ERP | Psychologcial placebo (stress management training) | Yes | March JS, Mulle K: OCD in children and adolescents: A cognitive-behavioral treatment manual. Guilford Press, 1998; Kozak M, Foa E: Mastery of obsessive-compulsive disorder: A cognitive-behavioral approach: Therapist guide. Oxford, England, Oxford University Press, 1997 | Yes | Therapy providers were trained to deliver both CBT and SMT. | Yes |
| 38 | O'Connor,1999 | Individual CBT | Care-as-usual | NR |  | NR |  | NR |
| 39 | O'Connor,2006 | Individual CBT | Pill placebo | Yes | Steketee G, ed. Treatment of obsessive–compulsive disorder. New York: The Guildord Press, 1993. Steketee G, ed. Overcoming obsessive–compulsive disorder: a behavioural and cognitive protocol for the treatment of OCD. Oakland: New Harbinger Publications, 1999. | Yes | All six therapists (S.R., S.G., M.-C.P., V.L., S.G. and P.D.) were CBT trained and supervised by one of the principal investigators. | Yes |
| 40 | Piacentini,2011 | Family-involved CBT | Psychologcial placebo (relaxation training) | Yes | Piacentini, J.; Langley, A.; Roblek, T. Cognitive-Behavioral Treatment of Childhood OCD: Therapist Guide. New York: Oxford University Press; 2007. Piacentini, J.; Langley, A.; Roblek, T. It’s Only a False Alarm: Child Workbook. New York: Oxford University Press; 2007. | Yes | Treatment was provided by doctoral-level psychologists and advanced clinical child psychology interns with specialty training in CBT for pediatric OCD | Yes |
| 41 | Russell,2013 | Individual ERP | Psychologcial placebo (anxiety management) | Yes | Russell AJ, Mataix-Cols D, Anson MA, Murphy DGM. Psychological treatment for obsessive compulsive disorder in people with autism spectrum disorders – a pilot study. Psychother Psychosom 2009;78:59–61. Attwood T. Modifications to cognitive behaviour therapy to accommodate the cognitive profile of people with Asperger’s syndrome. 1999. Available at: http://www.tonyattwood. com/paper2.htm. Anderson S, Morris J.Cognitive behaviour therapy for peoplewith Asperger syndrome. Behav Cogn Psychother 2006;34:293–303. | Yes | The treating therapists were all clinical psychologists (n=4) trained within a cognitive behavioral framework who had extensive experience in treating OCD in both young people and adults. | Yes |
| 42 | Russman Block,2023 | Individual ERP | Psychologcial placebo (stress management training) | Yes | March J, Mulle K: OCD in Children and Adolescents: A Cognitive-Behavioral Treatment Manual. New York, Guilford Press, 1998. Foa E, Kozak M: Mastery of obsessive-compulsive disorder: A cognitive-behavioral approach therapist guide. Oxford University Press, 2004 | Yes | Both conditions were standardized using written manuals on which therapists were trained and supervised (available on request). | Yes |
| 43 | Storch,2011 | Family-involved CBT | Waitlist | Yes | Pediatric OCD Treatment Study, 2004. Cognitive-behavior therapy, sertraline, and their combination for children and adolescents with obsessive-compulsive disorder: the pediatric OCD Treatment Study randomized controlled trial. Journal of the American Medical Association 292, 1969–1976. | Yes | Therapists were doctoral students in clinical psychology with at least 1 year of applied experience with CBT for OCD. | Yes |
| 44 | Schneider,2015 | Unguided self-help CT | Waitlist | Yes | Korrelboom, K. (2004). Behandelprotocol voor contraconditionering bij obsessies (Dutch) [Treatmet manual for counterconditioning with obsessions]. Unpublished manual. | Not applicable | | Yes |
| 45 | POTS,2004 | Individual CBT | Pill placebo | Yes | March J, Mulle K. OCD in Children and Adolescents: ACognitive-Behavioral Treatment Manual.New York, NY: Guilford Press; 1998. | NR |  | NR |
| 46 | Twohig,2010 | Individual third-wave CBT | Psychologcial placebo (relaxation training) | Yes | Hayes, SC.; Strosahl, KD.; Wilson, KG. Acceptance and Commitment Therapy: An experiential approach to behavior change. Guilford Press; New York: 1999. Bernstein, DA.; Borkovec, TD.; Hazlett-Stevens, H. New directions in progressive relaxation training: A guidebook for helping professionals. Praeger; New York: 2000. | Yes | All therapists and assessors were advanced graduate students in clinical psychology | Yes |
| 47 | Thompson-Hollands,2015 | Family-involved ERP |  | Yes | A treatment manual for the BFI was developed for the purposes of the present study | Yes | highly experienced clinicians | Yes |
|  |  | Individual ERP |  |  |  |  |  |  |
| 48 | van-Balkom,1998 | Individual CT | Waitlist | Yes | van Oppen P, Arntz A (1994) Cognitive therapy for obsessive compulsive disorder. Behav Res Ther 32:79-87. Hoogduin CAL, Hoogduin WA (1984) The out-patient treatment of patients with an obsessive-compulsive disorder. Behav Res Ther 22:455-460 | Yes | All therapists had experience with behavioral treatment for OCD and received training in cognitive therapy. | Yes |
|  |  | Individual ERP |  |  |  |  |  |  |
| 49 | Whittal,2010 | Individual CT | Psychologcial placebo (stress management training) | Yes | Rachman, S. (2003). The treatment of obsessions. Oxford: Oxford University Press. | Yes | Postdoctoral fellows with experience in the treatment of anxiety and OCD | Yes |
| 50 | Wilhelm,2009 | Individual CT | Waitlist | Yes | Wilhelm, S.; Steketee, G. Cognitive therapy for obsessive-compulsive disorder: A guide for professionals. Oakland, CA: New Harbinger Publications; 2006. | Yes | Treatment was administered by advanced doctoral students in psychology or by postdoctoral clinicians in training. | Yes |
| 51 | Williams,2010 | Individual CBT | Waitlist | Yes | Salkovskis PM (1998) Psychological approaches to the understanding of obsessional problems. In: Swinson RP, Antony MM, Rachman SJ, Richter MA (eds) Obsessive-compulsive disorder: theory, research and treatment. Guilford, New York | Yes | the therapists for the trial (TW, HW, ST) were clinical psychologists employed by the National Health Service (NHS) in England to work in community child and adolescent mental health clinics. | Yes |
| 52 | Wootton,2013 | Guided self-help CBT | Waitlist | Yes | The remote protocol used in the study (The OCD Course) was developed by the first three authors and contains 5 Lessons, designed to be read by participants over 8 weeks | Yes | The first author (BMW), a Clinical Psychologist, provided all clinical contact with participants (telephone calls, emails, and diagnostic interviews) and collated all the data. | Yes |
| 53 | Wolters,2016 | Individual CBT | Waitlist | Yes | De Haan, E., & Wolters, L. H. (2009). Behandeling van de dwangstoornis bij kinderen en adolescenten. Met het cognitief-gedragstherapeutisch protocol Bedwing je dwang. Houten: Bohn Stafleu van Loghum. | Yes | CBT was delivered by master level clinicians certified as cognitive behavioral therapists and experienced in treating OCD in children | Yes |
| 54 | Wu,2023 | Group CBT | Care-as-usual | Yes | Fan Q, Gao R, Bai Y, Sun Y. Group Cognitive Behavioral Therapy of OCD - A Treatment Manual. Shanghai: Shanghai Jiao Tong University Press; 2020. | Yes | The CBGT group was headed by a nationally registered therapist who had received systematic training. | Yes |
|  |  | Guided self-help CBT |  |  |  |  |  |  |
| 55 | Zhang,2021 | Group third-wave CBT | Psychologcial placebo (psychoeducation) | Yes | Didonna F, Lanfredi M, Xodo E, Ferrari C, Rossi R, Pedrini L. Mindfulnessbased cognitive therapy for obsessive-compulsive disorder: a pilot study. J Psychiatr Pract. (2019) 25:156–70. doi: 10.1097/PRA.0000000000000377 | Yes | Psychotherapists or psychiatrists specialized to OCD treatment; Trained by the founder of MBCT for OCD (Fabrizio Didonna) and one of the founders of MBCT (Mark Williams) to ensure treatment fidelity | NR |
| 56 | Zejuan-Fu,2016 | Individual CBT | Care-as-usual | NR |  | NR |  | NR |
| 57 | Hollmann,2022 | Remote-delivery CBT | Waitlist | Yes | Wewetzer G, Wewetzer C. Zwangsstörung bei Kindern und Jugendlichen – EinTherapiemanual. Göttingen: Hogrefe (2019). | Yes | The treatment providers were licensed psychotherapists with several years of professional experience and expertise in OCD in childhood and adolescence. They received supervision from the therapeutic head of study during weekly team meetings. | Yes |
| 58 | Turner,2014 | Individual CBT |  | Yes | Turner C, Heyman I, Futh A, Lovell K. A pilot study of telephone cognitive-behavioural therapy for obsessive-compulsive disorder in young people. Behav Cogn Psychother. 2009;37:469-474. Mataix-Cols D, Turner C, Monzani B, et al. Cognitive-behavioural therapy with post-session D-cycloserine augmentation for paediatric obsessive-compulsive disorder: pilot randomised controlled trial. Br J Psychiatry. 2014;204:77-78. | Yes | all treating therapists received supervision by more senior clinical psychologists who were specialists in CBT for OCD. | Yes |
|  |  | Remote-delivery CBT |  |  |  |  |  |  |
| 59 | Vogel,2014 | Remote-delivery ERP | Waitlist | NR |  | Yes | Therapists included one psychiatrist, one psychiatric nurse, and four psychologists, all experienced with ERP for OCD. | NR |
|  |  | Unguided self-help ERP |  |  |  |  |  |  |
| 60 | Tolin,2007 | Indiviudal ERP |  | Yes | Foa, E. B., & Kozak, M. J. (1997b). Mastery of obsessivecompulsive disorder: A cognitive-behavioral approach (therapist guide). New York: Oxford University Press. | Yes | Therapists included one psychiatrist, one psychiatric nurse, and four psychologists, all experienced with ERP for OCD. | Yes |
|  |  | Unguided self-help ERP |  |  |  |  |  |  |
| 61 | Lovell,2006 | Indiviudal CBT |  | Yes | Consistency oftreatment was maintained by therapist manuals, | Yes | Treatment was delivered by two trained and experienced cognitive behaviour therapists (one therapist at each site delivered both forms oftreatment) | Yes |
|  |  | Remote-delivery CBT |  |  |  |  |  |  |

**Appendix F. Assessment of risk of bias**

Abbreviations: SG, sequence generation [positive or negative (negative includes unclear)]; AC, allocation concealment [positive or negative (negative includes unclear)]; IOD, incomplete outcome data [positive or negative (negative includes unclear)]; BA, Blinded assessment [positive or negative (negative includes unclear)]; SOR, selective outcome reporting [positive or negative (negative includes unclear)]

Low risk of bias: The study is judged to be at low risk of bias for all domains for this result.

Some concerns: The study is judged to raise some concerns in at least one domain for this result, but not to be at high risk of bias for any domain.

High risk of bias: The study is judged to be at high risk of bias in at least one domain for this result. Or the study is judged to have some concerns for multiple (three) domains in the result.

**Appendix G. Pairwise meta-analyses of CBT delivery formats**

| **CBT format Comparison** | **N** | **SMD** | **95% CI** | ***I^2^*** | **95% CI** | **tau^2^** |
| --- | --- | --- | --- | --- | --- | --- |
| Individual vs |  |  |  |  |  |  |
| - Remote-delivery | 2 | -0.13 | [-0.46, 0.21] | 0 | - | - |
| - Group | 3 | -0.14 | [-0.46, 0.18] | 0 | [0, 89.6] | 0 |
| - Guided self-help | 1 | -0.32 | [-0.78, 0.14] | - | - |  |
| - Unguided self-help | 3 | -0.37 | [-0.64, -0.09] | 0 | - | 0 |
| - Family-involved | 2 | 0.58 | [-0.02, 1.17] | 0 | - | 0 |
| - Waitlist | 11 | -1.36 | [-1.80, -0.92] | 73% | [49.73, 85.05] | 0.39 |
| - Care-as-usual | 2 | -1.28 | [-2.37, -0.19] | 69% | - | 0.45 |
| - Psychological placebo | 7 | -0.91 | [-1.17; -0.66] | 47% | [0, 77.77] | 0.05 |
| - Pill placebo | 2 | -1.12 | [-1.67, -0.58] | 13% | - | 0.03 |
| Remote-delivery vs |  |  |  |  |  |  |
| - Unguided self-help | 1 | -2.06 | [-3.16, -0.96] |  |  |  |
| - Waitlist | 2 | -1.82 | [-2.49, -1.15] | 24% | - | 0.07 |
| Group vs |  |  |  |  |  |  |
| - Guided self-help | 1 | -0.02 | [-0.53, 0.49] | - | - | - |
| - Waitlist | 6 | -0.81 | [-1.24, -0.37] | 73% | [37.81, 88.22] | 0.22 |
| - Care-as-usual | 1 | -0.56 | [-1.07, -0.05] | - | - | - |
| - Psychological placebo | 1 | -0.27 | [-0.8, 0.25] | - | - | - |
| Guided self-help vs |  |  |  |  |  |  |
| - Unguided self-help | 1 | -0.33 | [-0.78, 0.13] | - | - | - |
| - Waitlist | 3 | -1.07 | [-1.5; -0.65] | 10% | [0, 90.64] | 0.01 |
| - Care-as-usual | 1 | -0.52 | [-1.02, -0.03] | - | - | - |
| - Psychological placebo | 2 | -0.81 | [-1.36, -0.25] | 79% | - | 0.13 |
| Unguided self-help vs |  |  |  |  |  |  |
| - Waitlist | 6 | -0.53 | [-0.75, -0.3] | 0 | [0, 74.62] | 0 |
| - Psychological placebo | 3 | -0.94 | [-1.83, -0.05] | 89% | [70.04, 95.99] | 0.49 |
| Family-involved vs |  |  |  |  |  |  |
| - Waitlist | 4 | -1.27 | [-1.94, -0.61] | 77% | [36.37, 91.49] | 0.34 |
| - Care-as-usual | 2 | -1.64 | [-2.3, -0.98] | 0 | - | 0 |
| - Psychological placebo | 3 | -0.73 | [-1.41, -0.05] | 81% | [38.94, 93.81] | 0.29 |
| Time-intensive vs |  |  |  |  |  |  |
| - Care-as-usual | 1 | -0.90 | [-1.61, -0.18] | - | - | - |
| - Psychological placebo | 1 | -2.89 | [-4.25, -1.53] | - | - | - |
| - Pill placebo | 1 | -1.52 | [-2.22, -0.83] | - | - | - |

Abbreviations: N, number of trials; SMD, standard mean difference; CI, confidence interval

**Appendix H. Rank of CBT delivery formats by SUCRA**

| **CBT delivery format** | **Effectiveness** | **Acceptability** |
| --- | --- | --- |
| Time-intensive | 92% | 41% |
| Remote-delivery | 87% | 81% |
| Family-involved | 80% | 61% |
| Individual | 76% | 54% |
| Guided self-help | 59% | 59% |
| Group | 52% | 74% |
| Unguided self-help | 42% | 15% |
| Psychological placebo | 25% | 59% |
| Care-as-usual | 15% | 28% |
| Pill placebo | 14% | 25% |
| Waitlist | 7% | 52% |


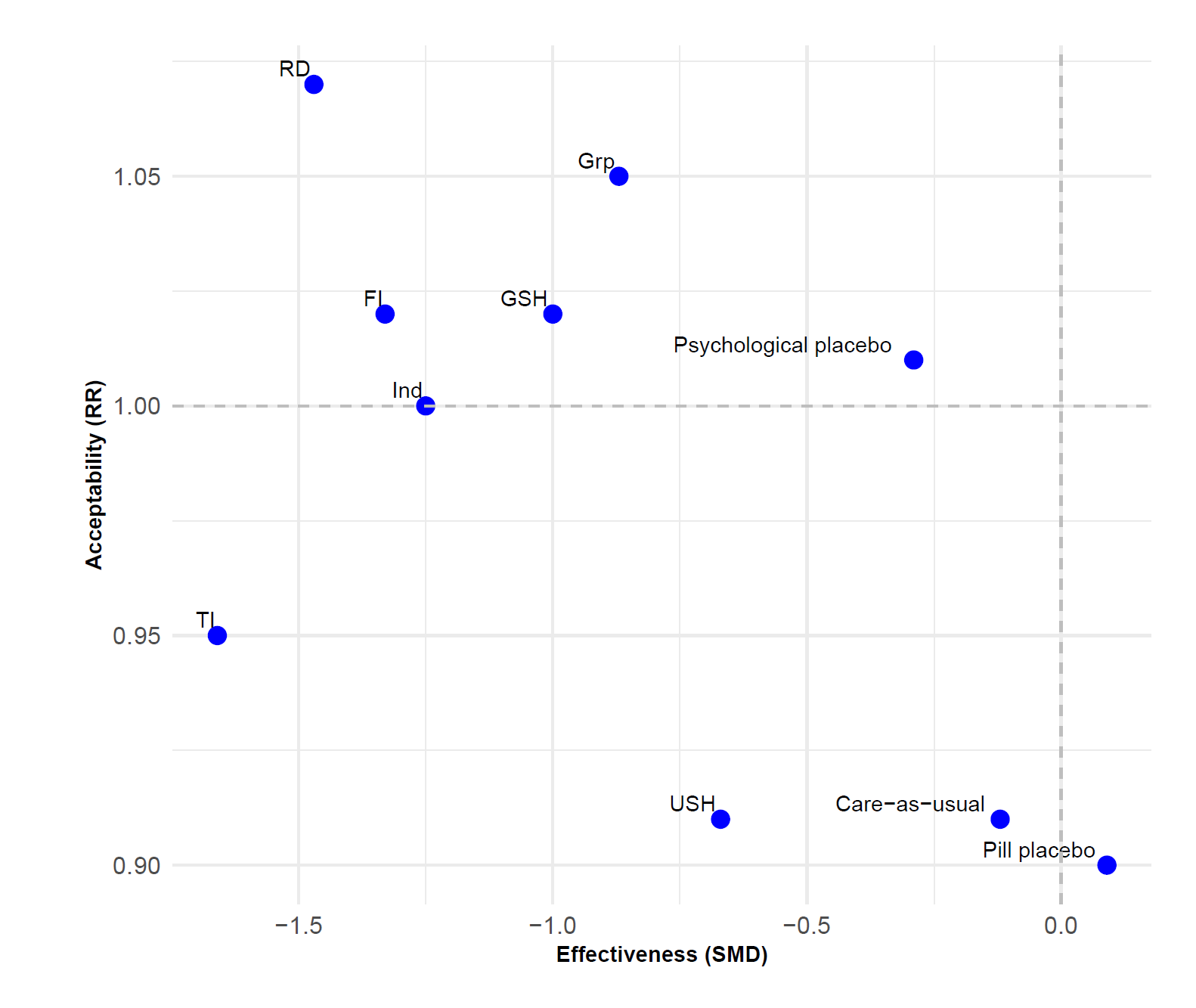


The Scatter Plot of Effectiveness and Acceptability of various CBT formats in comparison to a waitlist. Standardized mean difference (SMD) lower than 0 represent greater effect. Relative Risk (RR) greater than 1 represents higher acceptability. TI, time-intensive; RD, remote-delivery; FI, family-involved; Ind, individual; GSH, guided self-help; Grp, group; USH, unguided self-help. The Scatter Plot of Effectiveness and Acceptability of CBT formats. TI, time-intensive; RD, remote-delivery; FI, family-involved; Ind, individual; GSH, guided self-help; Grp, group; USH, unguided self-help.

**Appendix I. GRADE appraisal (CINeMA)**

The analysis of the certainty of the evidence was performed with the online application CINeMA, which follows the principles of the GRADE methodology. The following criteria were applied:

- Within-study bias: the “overall” risk of bias of each study was calculated as follows:

(a) **LOW RISK** if all domains were judged as “low risk”;

(b) **SOME CONCERNS** if one domain was judged as “some concerns”

(c) **HIGH RISK** if at least one domain had “high risk” or multiple domains had some concerns (3 domains with some concerns)

For each comparison, the histogram was interpreted according to a “Average risk of bias” rule;

- Across-studies bias was considered “undetected” when was not possible to evaluate the risk of publication bias;
- Imprecision: an effect size between 0.2 was considered as clinically important size of effect;
- Heterogeneity: an effect size of 0.2 was considered as clinically important size of effect;
- Incoherence: for all the comparisons for which only a direct or indirect estimation was available (Inconsistency measures: Not applicable) we reported “some concerns”.

Final report

**Appendix J. Transitivity assessment: characteristics of included studies across different comparisons**

Abbreviations: Ind, individual; RD, remote-delivery; Grp, group; GSH, guided self-help; USH, unguided self-help; FI, family-involved; TI, time-intensive; WL, waitlist; CAU, care-as-usual; psy P, psychological placebo; pill P, pill placebo; OCD_BSS, weighted baseline OCD symptom severity; % Female, proportion of female; % Med_treat: proportion of participants using psychiatric medicine in the treatment group; Recruit, the methods of recruiting participants (Clin, clinical; Com, community; Oth, not belong to clinical or community); Profess, the treatment was delivered by professionals; Fidelity, the trialists performed fidelity checks of the interventions; Manual, treatment manual/protocol was used

**Appendix K. Inconsistency check – local and global test**

Local test: the Net Heat plot


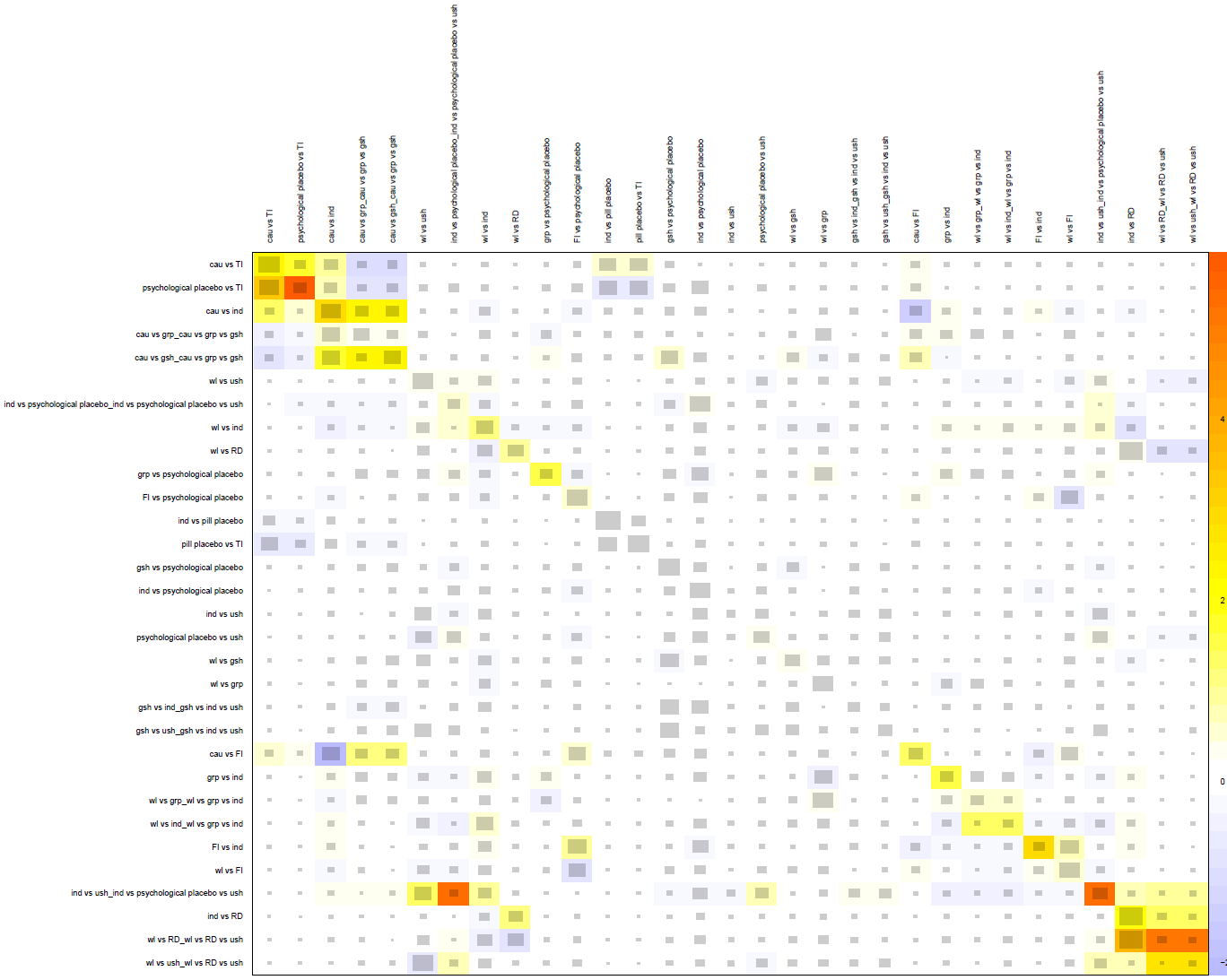


Abbreviations: ind, individual; grp, group; gsh, guided self-help; ush, unguided self-help; wl, waitist; cau, care-as-usual

The quadratic heatmap in which each design in a row is compared to the other designs (in the columns). The rows and columns signify specific designs, not individual treatment comparisons in the network:

- Gray boxes: how important a treatment comparison is for the estimation of another treatment comparison. The bigger the box, the important the treatment comparison.
- Colored backgrounds: signify the inconsistency of the design in the row that can be attributed to the design in a column. Field color can range from a deep red (strong inconsistency) to blue (indicate that evidence from this design supports evidence in the row).

Global test: Net Splitting


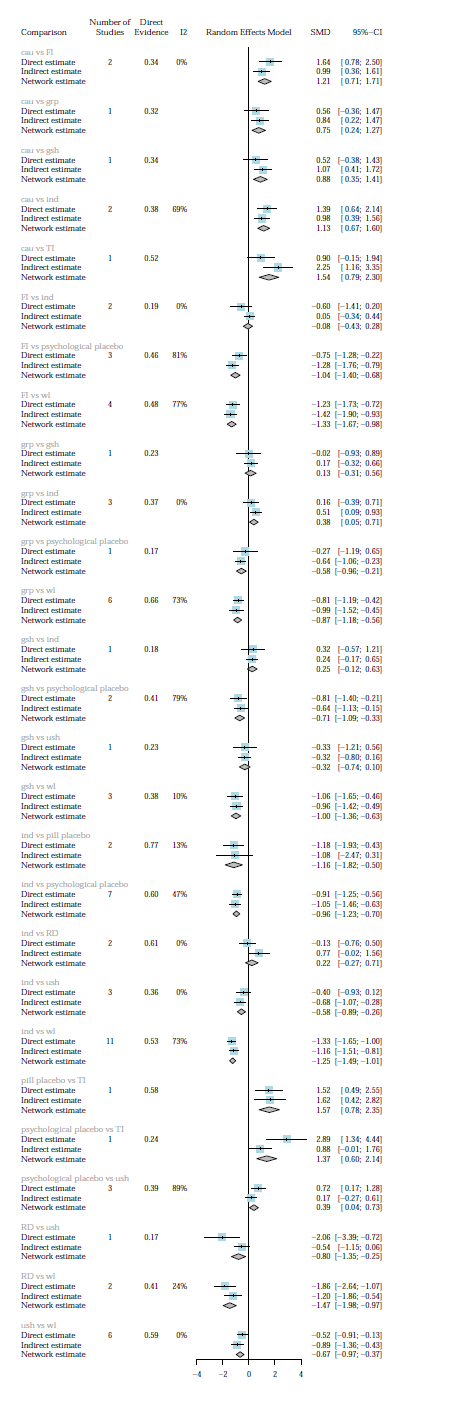


**Appendix L. Sensitivity analysis: Outliers excluded**

**Pairwise meta-analyses**

| **Format Comparison** | **No.** | **SMD** | **95% CI** | ***I^2^* Statistic** | | **tau^2^** |
| --- | --- | --- | --- | --- | --- | --- |
| Individual VS |  |  |  | |  |  |
| - Remote-delivery | 2 | -0.13 | [-0.46; 0.21] |  | |  |
| - Group | 3 | -0.14 | [-0.46, 0.18] | 0 | | 0 |
| - Guided self-help | 1 | -0.32 | [-0.78, 0.14] | - | | - |
| - Unguided self-help | 3 | -0.37 | [-0.64; -0.09] | 0 | | 0 |
| - Family-involved | 2 | 0.58 | [-0.02; 0.17] | 0 | | 0 |
| - Waitlist | 10 | -1.2 | [-1.58; -0.83] | 59% | | 0.21 |
| - Care-as-usual | 2 | -1.28 | [-2.37, -0.19] | 69% | | 0.45 |
| - Psychological placebo | 7 | -0.91 | [-1.17; -0.66] | 47% | | 0.05 |
| - Pill placebo | 2 | -1.12 | [-1.67, -0.58] | 13% | | 0.03 |
| Remote-delivery vs |  |  |  |  | |  |
| - Unguided self-help | 1 | -2.06 | [-3.16; -0.96] |  | |  |
| - Waitlist | 2 | -1.82 | [-2.49; -1.15] | 24% | | 0.07 |
| - Group VS |  |  |  |  | |  |
| - Guided self-help | 1 | -0.02 | [-0.53, 0.49] | - | | - |
| - Waitlist | 6 | -0.81 | [-1.24, -0.37] | 73% | | 0.22 |
| - Care-as-usual | 1 | -0.56 | [-1.07, -0.05] | - | | - |
| - Psychological placebo | 1 | -0.27 | [-0.8, 0.25] | - | | - |
| Guided self-help VS |  |  |  |  | |  |
| - Unguided self-help | 1 | -0.33 | [-0.78, 0.13] |  | |  |
| - Waitlist | 3 | -1.07 | [-1.5; -0.65] | 10% | | 0.01 |
| - Care-as-usual | 1 | -0.52 | [-1.02, -0.03] | - | | - |
| - Psychological placebo | 2 | -0.81 | [-1.36, -0.25] | 79% | | 0.13 |
| Unguided self-help VS |  |  |  |  | |  |
| - Waitlist | 6 | -0.53 | [-0.75, -0.3] | 0 | | 0 |
| - Psychological placebo | 3 | -0.94 | [-1.83, -0.05] | 89% | | 0.49 |
| Family-involved VS |  |  |  |  | |  |
| - Waitlist | 4 | -1.27 | [-1.94, -0.61] | 77% | | 0.34 |
| - Care-as-usual | 2 | -1.64 | [-2.3, -0.98] | 0 | | 0 |
| - Psychological placebo | 3 | -0.73 | [-1.41, -0.05] | 81% | | 0.29 |
| Time-intensive VS |  |  |  |  | |  |
| - Care-as-usual | 1 | -0.9 | [-1.61, -0.18] | - | | - |
| - Psychological placebo | 1 | -2.89 | [-4.25, -1.53] | - | | - |
| - Pill placebo | 1 | -1.52 | [-2.22, -0.83] | - | | - |

**Network meta-analyses**

**Appendix M. Sensitivity analysis: Studies with comorbidities (when participants exhibiting at least one shared type of mental disorder) excluded**

**Pairwise meta-analyses**

**Network meta-analyses**

| **Format Comparison** | **No.** | **SMD** | **95% CI** | ***I^2^* Statistic** | **tau^2^** |
| --- | --- | --- | --- | --- | --- |
| Individual VS |  |  |  |  |  |
| - Remote-delivery | 1 | -0.17 | [-0.63; 0.29] |  |  |
| - Group | 3 | -0.14 | [-0.46, 0.18] | 0 | 0 |
| - Guided self-help | 1 | -0.32 | [-0.78, 0.14] | - |  |
| - Unguided self-help | 3 | -0.37 | [-0.64; -0.09] | 0 | 0.47 |
| - Family-involved | 2 | 0.58 | [-0.02; 0.17] | 0 | 0 |
| - Waitlist | 9 | -1.29 | [-1.73; -0.84] | 65% | 0.29 |
| - Care-as-usual | 2 | -1.28 | [-2.37, -0.19] | 69% | 0.45 |
| - Psychological placebo | 5 | -0.94 | [-1.18; -0.69] | 37% | 0.03 |
| - Pill placebo | 2 | -1.12 | [-1.67, -0.58] | 13% | 0.03 |
| Remote-delivery vs |  |  |  |  |  |
| - Unguided self-help | 1 | -2.06 | [-3.16; -0.96] | - | - |
| - Waitlist | 2 | -1.82 | [-2.49; -1.15] | 24% | 0.07 |
| - Group VS |  |  |  |  |  |
| - Guided self-help | 1 | -0.02 | [-0.53, 0.49] |  |  |
| - Waitlist | 6 | -0.81 | [-1.24, -0.37] | 73% | 0.22 |
| - Care-as-usual | 1 | -0.56 | [-1.07, -0.05] | - | - |
| - Psychological placebo | 1 | -0.27 | [-0.8, 0.25] | - | - |
| Guided self-help VS |  |  |  |  |  |
| - Unguided self-help | 1 | -0.33 | [-0.78, 0.13] |  |  |
| - Waitlist | 3 | -1.07 | [-1.5; -0.65] | 10% | 0.01 |
| - Care-as-usual | 1 | -0.52 | [-1.02, -0.03] | - | - |
| - Psychological placebo | 2 | -0.81 | [-1.36, -0.25] | 79% | 0.13 |
| Unguided self-help VS |  |  |  |  |  |
| - Waitlist | 6 | -0.53 | [-0.75, -0.3] | 0 | 0 |
| - Psychological placebo | 3 | -0.94 | [-1.83, -0.05] | 89% | 0.49 |
| Family-involved VS |  |  |  |  |  |
| - Waitlist | 4 | -1.27 | [-1.94, -0.61] | 77% | 0.34 |
| - Care-as-usual | 2 | -1.64 | [-2.3, -0.98] | 0 | 0 |
| - Psychological placebo | 3 | -0.73 | [-1.41, -0.05] | 81% | 0.29 |
| Time-intensive VS |  |  |  |  |  |
| - Care-as-usual | 1 | -0.9 | [-1.61, -0.18] | - | - |
| - Psychological placebo | 1 | -2.89 | [-4.25, -1.53] | - | - |
| - Pill placebo | 1 | -1.52 | [-2.22, -0.83] | - | - |

**Appendix N. Sensitivity analysis: Only studies with low risk of bias**

**Pairwise meta-analyses**

| **Format Comparison** | **No.** | **SMD** | **95% CI** | ***I^2^* Statistic** | **tau^2^** |
| --- | --- | --- | --- | --- | --- |
| Individual VS |  |  |  |  |  |
| - Remote-delivery | 2 | -0.13 | [-0.46; 0.21] |  |  |
| - Guided self-help | 1 | -0.32 | [-0.78; 0.14] |  |  |
| - Unguided self-help | 1 | -0.58 | [-1.06; -0.10] |  |  |
| - Waitlist | 2 | -0.77 | [-1.20; -0.33] | 0 | 0 |
| - Psychological placebo | 2 | -0.93 | [-1.26; -0.60] | 0 | 0 |
| Remote-delivery vs |  |  |  |  |  |
| - Waitlist | 1 | -1.61 | [-2.20; -1.03] |  |  |
| Group VS |  |  |  |  |  |
| - Waitlist | 1 | -1.18 | [-1.81; -0.56] |  |  |
| Guided self-help VS |  |  |  |  |  |
| - Unguided self-help | 1 | -0.33 | [-0.78; 0.13] |  |  |
| - Waitlist | 1 | -0.92 | [-1.67; -0.16] |  |  |
| - Psychological placebo | 1 | -1.11 | [-1.53; -0.69] |  |  |
| Unguided self-help VS |  |  |  |  |  |
| - Waitlist | 1 | -0.24 | [-0.73; 0.25] |  |  |
| Family-involved VS |  |  |  |  |  |
| - Waitlist | 1 | -0.68 | [-1.18; -0.19] |  |  |
| - Care-as-usual | 2 | -1.64 | [-2.30; -0.98] |  |  |
| - Psychological placebo | 2 | -0.85 | [-1.82; 0.11] | 86% | 0.42 |
| Time-intensive VS |  |  |  |  |  |
| - Care-as-usual | 1 | -0.9 | [-1.61; -0.18] |  |  |

**Network meta-analyses**

**Appendix O. Sensitivity analysis: CBT studies combining both cognitive and behavioural therapeutic techniques**

**Pairwise meta-analyses**

| **Format Comparison** | **No.** | **SMD** | **95% CI** | ***I^2^* Statistic** | **tau^2^** |
| --- | --- | --- | --- | --- | --- |
| Individual VS |  |  |  |  |  |
| - Remote-delivery | 1 | -0.17 | [-0.63; 0.29] |  |  |
| - Group | 3 | -0.14 | [-0.46; 0.18] | 0 | 0 |
| - Guided self-help | 1 | -0.32 | [-0.78; 0.14] | |  |
| - Unguided self-help | 1 | -0.58 | [-1.06; -0.10] | |  |
| - Waitlist | 7 | -1.11 | [-1.57; -0.65] | 67% | 0.25 |
| - Care-as-usual | 2 | -1.28 | [-2.37; -0.19] | 69% | 0.45 |
| - Pill placebo | 2 | -1.12 | [-1.67; -0.58] | 13% | 0.03 |
| Remote-delivery vs |  |  |  |  |  |
| - Waitlist | 1 | -1.61 | [-2.20; -1.03] | |  |
| Group VS |  |  |  |  |  |
| - Guided self-help | 1 | -0.02 | [-0.53; 0.49] | |  |
| - Waitlist | 5 | -1.02 | [-1.31; -0.74] | 20% | 0.02 |
| - Care-as-usual | 1 | -0.56 | [-1.07; -0.05] | |  |
| Guided self-help VS |  |  |  |  |  |
| - Unguided self-help | 1 | -0.33 | [-0.78; 0.13] | |  |
| - Waitlist | 2 | -1.22 | [-1.74; -0.70] | 11% | 0.02 |
| - Care-as-usual | 1 | -0.52 | [-1.02; -0.03] | |  |
| - Psychological placebo | 2 | -0.81 | [-1.36; -0.25] | 79% | 0.13 |
| Unguided self-help VS |  |  |  |  |  |
| - Waitlist | 1 | -0.74 | [-1.27; -0.20] | |  |
| Family-involved VS |  |  |  |  |  |
| - Waitlist | 4 | -1.27 | [-1.94; -0.61] | 77% | 0.34 |
| - Psychological placebo | 3 | -0.73 | [-1.41; -0.05] | 81% | 0.29 |
| Time-intensive VS |  |  |  |  |  |
| - Care-as-usual | 1 | -0.9 | [-1.61; -0.18] | |  |

**Network meta-analyses**

**Appendix P. Long-term effects at 3 – 12 month**

**Pairwise meta-analysis**

**Network meta-analyses**

| **Format Comparison** | **No.** | **SMD** | **95% CI** | ***I^2^* Statistic** | **tau^2^** |
| --- | --- | --- | --- | --- | --- |
| Individual VS |  |  |  |  |  |
| - Remote-delivery | 2 | -0.12 | [-0.45; 0.22] |  |  |
| - Group | 1 | 0.03 | [-0.45; 0.51] |  |  |
| - Guided self-help | 1 | -0.35 | [-0.82; 0.12] |  |  |
| - Unguided self-help | 2 | -0.52 | [-0.92; -0.12] |  |  |
| - Family-involved | 1 | 0.44 | [-0.31; 1.19] |  |  |
| - Waitlist | 1 | -2.92 | [-3.82; -2.02] |  |  |
| - Psychological placebo | 4 | -0.5 | [-0.89; -0.11] | 45% | 0.07 |
| Group VS |  |  |  |  |  |
| - Psychological placebo | 1 | 0.36 | [-0.17; 0.88] |  |  |
| Guided self-help VS |  |  |  |  |  |
| - Unguided self-help | 1 | -0.19 | [-0.64; 0.26] |  |  |
| Unguided self-help VS |  |  |  |  |  |
| - Waitlist | 1 | -0.28 | [-1.02; 0.47] |  |  |
| - Psychological placebo | 1 | 0.06 | [-0.29; 0.40] |  |  |
